# Extensive transcriptomic and epigenomic remodelling occurs during *Arabidopsis thaliana* germination

**DOI:** 10.1186/s13059-017-1302-3

**Published:** 2017-09-15

**Authors:** Reena Narsai, Quentin Gouil, David Secco, Akanksha Srivastava, Yuliya V. Karpievitch, Lim Chee Liew, Ryan Lister, Mathew G. Lewsey, James Whelan

**Affiliations:** 1 ARC Centre of Excellence in Plant Energy Biology, Department of Animal, Plant and Soil Sciences, School of Life Sciences, La Trobe University, Melbourne, VIC 3086 Australia; 2 Centre for AgriBioscience, Department of Animal, Plant and Soil Sciences, School of Life Sciences, La Trobe University, Melbourne, VIC 3086 Australia; 3 ARC Centre of Excellence in Plant Energy Biology, The University of Western Australia, Perth, WA 6009 Australia; 4 Harry Perkins Institute of Medical Research, Perth, WA 6009 Australia

**Keywords:** Arabidopsis, Germination, RNA-seq, Alternative splicing, DNA methylation, Small RNA, Transcription factor

## Abstract

**Background:**

Seed germination involves progression from complete metabolic dormancy to a highly active, growing seedling. Many factors regulate germination and these interact extensively, forming a complex network of inputs that control the seed-to-seedling transition. Our understanding of the direct regulation of gene expression and the dynamic changes in the epigenome and small RNAs during germination is limited. The interactions between genome, transcriptome and epigenome must be revealed in order to identify the regulatory mechanisms that control seed germination.

**Results:**

We present an integrated analysis of high-resolution RNA sequencing, small RNA sequencing and MethylC sequencing over ten developmental time points in *Arabidopsis thaliana* seeds, finding extensive transcriptomic and epigenomic transformations associated with seed germination. We identify previously unannotated loci from which messenger RNAs are expressed transiently during germination and find widespread alternative splicing and divergent isoform abundance of genes involved in RNA processing and splicing. We generate the first dynamic transcription factor network model of germination, identifying known and novel regulatory factors. Expression of both microRNA and short interfering RNA loci changes significantly during germination, particularly between the seed and the post-germinative seedling. These are associated with changes in gene expression and large-scale demethylation observed towards the end of germination, as the epigenome transitions from an embryo-like to a vegetative seedling state.

**Conclusions:**

This study reveals the complex dynamics and interactions of the transcriptome and epigenome during seed germination, including the extensive remodelling of the seed DNA methylome from an embryo-like to vegetative-like state during the seed-to-seedling transition. Data are available for exploration in a user-friendly browser at https://jbrowse.latrobe.edu.au/germination_epigenome.

**Electronic supplementary material:**

The online version of this article (doi:10.1186/s13059-017-1302-3) contains supplementary material, which is available to authorized users.

## Background

Seeds are essential for crop productivity and are an important part of our diet. They can remain dormant for years before becoming highly metabolically active as the seed germinates and transitions into a seedling. Seeds constantly perceive specific cues, such as the presence of water, light, temperature and nutrients, which trigger molecular responses and enable germination to progress [1]. These responses include hormone signalling, among which the antagonistic interactions between abscisic acid (ABA) and gibberellic acid (GA) are most intensively studied [1, 2]. Alterations in the levels of microRNAs (miRNAs), transcripts or DNA methylation affect seed dormancy, seed viability, germination and seedling development [3–7]. These layers of regulation interact extensively, with the result that a complex network of inputs contributes to germination and successful seedling establishment.

Microarray-based transcriptomic studies in various plants have described global changes in the cellular messenger RNA (mRNA) population and hormone interactions during seed germination, such as ABA regulation of the germination transcriptome [8–12]. They have also enabled network modelling of global transcriptional interactions in seeds (SeedNet) [13]. While highly informative, microarray platforms are limited to a defined set of probes and have lower sensitivity than current RNA-sequencing (RNA-seq) methods. Furthermore, RNA-seq enables discovery of unannotated loci specific to the developmental stage or tissue studied and allows the quantification of individual isoforms. Alternative splicing patterns may also be stage or tissue-specific and can influence mRNA stability or protein function [14–17]. For example, differential splicing occurs in *A. thaliana* (Arabidopsis) pollen and seedlings [15]. Alternative splicing expands the repertoire of transcripts derived from a genome: the latest genome annotation of Arabidopsis (Araport11) documents 48,359 transcripts corresponding to 27,655 genes.

RNA silencing is a mechanism for genome regulation and defence that targets transcripts and genomic loci using small RNAs (sRNAs) Sequence complementarity between the sRNA and the locus or transcript guides the RNA silencing machinery to its target. MiRNAs are a type of sRNA that regulate complementary transcripts by degradation or repression of translation [18]. They have a regulatory role in plant development [19] and under stress conditions [20]. For example, miRNA159 and miRNA160 interact with ABA/GA signalling pathways during seed germination in Arabidopsis [19, 21]. Small-interfering RNAs (siRNAs) of 23–24 nt are involved in the RNA-directed DNA methylation (RdDM) pathway, recruiting the de novo methyltransferase DOMAINS REARRANGED 2 (DRM2) to methylate cytosines in all contexts [22]. While 20–22-nt siRNAs primarily mediate post-transcriptional gene silencing through cleavage of their complementary targets, they are also able to direct DNA methylation [23]. DNA methylation influences chromatin structure and has a constitutive role in the transcriptional regulation of genes and repeats. DNA methylation profiles also display tissue specificity [24], are remodelled during plant sexual reproduction [25–27], and react to biotic and abiotic stresses [28–30]. DNA methylation is maintained through mitotic and meiotic replication by DNA methyltransferases with distinct sequence affinities. MET1 maintains CG methylation, CMT3 performs CHG methylation (where H is any nucleotide but G) and CMT2 methylates several CHH cytosine contexts to variable extents [31, 32]. During germination, DNA demethylation has been observed in pepper [33], wheat [34] and rice [35]. Loss-of-function mutations in genes involved in DNA methylation, such as *MET1*, and demethylation, such as *DEMETER*, result in embryo-defective phenotypes in Arabidopsis [7, 36], indicating that the regulation of DNA methylation in the seed is essential for normal development.

**Fig. 1:**
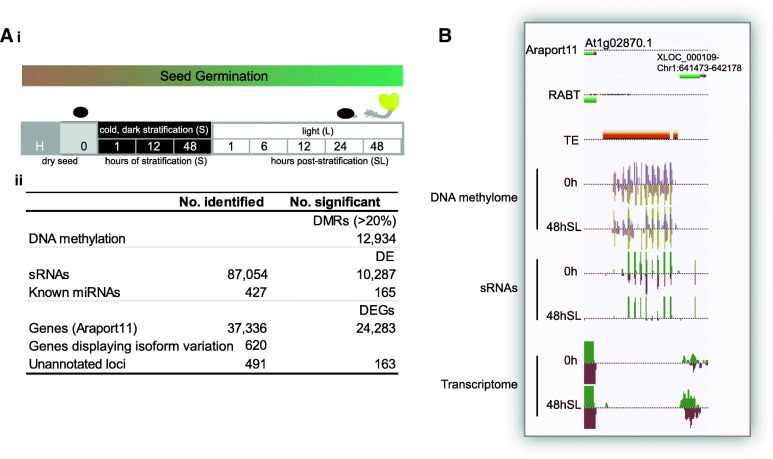
Overview of the extensive transcriptomic and epigenetic remodelling that occurs during seed germination. **a** (i) The time course examined in this study. Transcriptomes and sRNAs were analysed at all time points. H denotes freshly harvested seeds, collected before two weeks of dry, dark ripening. DNA methylation was analysed at 0 h (after ripening), 48 h S, 6 h SL, 24 h SL and 48 h SL. (ii) The number of differentially methylated regions (DMRs), sRNAs and genes that were identified and differentially expressed (DE) over germination are shown (as total numbers for all time points combined/compared). **b** An example of an unannotated DE locus (XLOC_000109), with nearby differential methylation and overlapping sRNAs, as shown in the AnnoJ genome browser

To reveal the molecular networks governing seed germination in Arabidopsis, we assayed genome-wide sites of DNA methylation (MethylC-seq), the transcriptome (RNA-seq) and the cellular sRNA population (sRNA-seq) over an extensive time course, from before seed desiccation through stratification and germination to post-germination (Fig. 1a(i)). We detected 50% more differentially expressed genes (DEG) during germination compared to previous studies [9, 10]. A total of 620 genes used isoforms differentially during germination (Fig. 1a(ii)), significantly expanding upon prior single gene studies during development [37, 38]. A total of 163 previously unannotated differentially expressed (DE) loci were identified during germination (Fig. 1a(ii)). Transcription factor (TF) regulatory network models confirmed the involvement of known germination regulators, such as ABI5 and ATHB5 [6, 39], and identified new TFs that may regulate specific stages of germination. We observed delayed germination in seven out of eight lines carrying mutations in TFs predicted to be important by the model. The germination transcriptome was disrupted by each of the eight TF mutations. Significant differential expression of miRNAs and siRNAs were detected during germination (Fig. 1a(ii)) and extensive epigenetic remodelling was observed between the seed and post-germinative seedling (Fig. 1a(ii)), with CHH hypomethylation detected at 12,654 loci. Our study provides an unprecedented view into the dynamics and interactions of the epigenome and transcriptome during seed germination. It also expands our knowledge of the complexity of direct TF-gene interaction and sets the foundation for a systems-level understanding. The specific regulators and miRNAs presented here are excellent candidates for manipulation to modify germination characteristics.

## Results

### Differential RNA splicing changes relative transcript isoform abundance during germination

The primary aim of our study was to determine the interactions between genome and epigenome during germination. Our approach was to measure RNA, sRNA and mC dynamics across a time series from dry seed to seedling (Fig. 1a(i, ii)) and to relate these to the major developmental transitions during germination. Furthermore, we aimed to identify TFs that regulate transcript abundance by integrating gene targeting data with the expression time series. This would enable us to increase systems-level understanding of the direct regulators of gene expression during germination, a notable gap in current knowledge. To facilitate visualisation, evaluation and reuse of our data, all transcriptomes, sRNA-omes, methylomes and useful annotations (TF binding peaks, sRNA loci, differentially methylated regions [DMRs]) are integrated in a JBrowse browser (https://jbrowse.latrobe.edu.au/germination_epigenome).

We observed seed swelling associated with water uptake following 48 h of stratification (S) then 12 h in the light (L; combined treatment termed 12 h SL) and radicle emergence occurred by 24 h SL. The rapid germination of these seeds is consistent with their expected lack of dormancy, given that they were harvested from plants grown at 22 °C [40, 41]. However, we stratified seeds in this experiment to reflect common lab germination procedures for Arabidopsis accession Col-0 (https://abrc.osu.edu/seed-handling). Over this time course, we first analysed the dynamics of transcript abundance during germination by whole-transcriptome RNA-seq (Fig. 1a(ii); Additional file 1: SD1). This enabled the identification of unannotated loci (Fig. 1a(ii) and b) and revealed over 24,283 DEGs during germination (Fig. 1a(ii); Additional file 1: SD1), a 50% increase over previous microarray studies where fewer than 16,000 DEGs were identified [9, 10]. We defined three clusters on the basis of their profiles using hierarchical clustering; grouping genes whose expression increased or decreased by the end of the time course or showed a transient peak during seed germination (Additional file 2: Figure S1). The gene ontology (GO) functional enrichments (http://geneontology.org/) in each cluster were consistent with previous studies [8–10]: light-related and root-related functions were enriched in the cluster of genes with highest expression in the seedling, RNA splicing and histone functions were enriched for genes with high expression in dry seed and genes encoding mitochondrial proteins and RNA-related functions were enriched among the transiently expressed genes (Additional file 2: Figure S1).

**Fig. 2:**
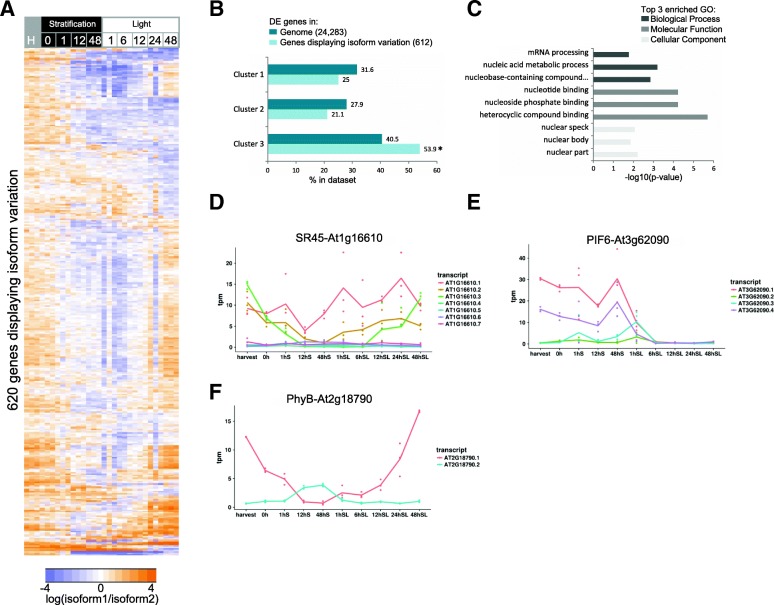
Alternative splicing of RNA processing genes occurs during germination. **a** *Heatmap* of the log ratios of isoform1/isoform2 for the 620 genes with at least two isoforms and average expression greater than 0.1 transcript per million (tpm). Only the genes that had a maximum isoform1/isoform2 ratio of > 2 and a minimum isoform1/isoform2 ratio of < 0.5 over the time course are shown. **b** Of the 620 genes displaying isoform variation, 612 were differentially expressed during germination. The proportion of these falling into the three clusters compared to the genome is shown (Cluster 1: increasing over time course, highest expression in seedling, Cluster 2: transiently peaking, Cluster 3: decreasing expression over time course with highest expression in seeds). **c** GO enrichment analysis showing the top three enriched categories. Examples of genes showing variations in isoform expression including (**d**) *SR45*, (**e**) *PIF6* and (**f**) *PhyB*. The *solid lines* represent the average of three replicates

We next analysed alternative splicing during germination to determine its contribution to transcriptome reprogramming. Relative abundance of isoforms from the same gene was positively correlated in the majority of cases. However, isoforms of 141 genes were anti-correlated (Pearson’s correlation coefficients below – 0.5), suggesting isoform usage may vary during germination (Additional file 2: Figure S2). We found that the hierarchy of primary and secondary isoforms was inverted for 620 genes during germination (their ratio of expression spanned a range outside of 0.5:2; Additional file 3: Table S1). Hierarchical clustering of these ratios showed that isoform variation was particularly distinct between dry seed and post-imbibition, suggestive of time-specific or tissue-specific regulation of alternative splicing during seed germination (Fig. 2a). Of the genes with isoform variation, 612 were also differentially expressed at the gene level and 54% of these belonged to cluster C3 (highest expression in seeds, then decreasing over germination—as defined in Additional file 2: Figure S1), which is significantly more than the expected percentage of genes in C3 compared to the total percentage in the genome (*p* < 0.05, Fig. 2b).

The highly dynamic alternative splicing during seed germination affected splicing regulators themselves. GO analysis revealed significant (*p* < 0.01) enrichment of genes involved in nucleotide/nucleoside binding, mRNA processing and nucleic acid metabolic processes (Fig. 2c). Interestingly, GO enrichment analysis indicated these were enriched in the nuclear speck, where splicing factors are known to be localised (Fig. 2c). A role for alternative splicing of phytochrome interacting factor 6 (*PIF6*, At3g62090) and serine-arginine-rich (SR) protein 45 (*SR45*, At1g16610) has been previously demonstrated [37, 38]. We found complex variations in total and relative abundance of their multiple isoforms (Fig. 2d, e). *SR45* encodes a key pre-mRNA splicing factor in Arabidopsis, the alternative splicing of which affects petal development and root growth during early seedling development [38]. SR45 regulates glucose and ABA signalling [42], with over 30% of all ABA signalling genes associated with or regulated by SR45 at the post-splicing level [43]. Alternative splicing of *PIF6* influences rates of ABA-dependent seed germination in Arabidopsis [37]. We found that phytochrome B (PhyB, At2g18790), which interacts with PIF6, also showed isoform variation during seed germination: isoform At2g18790.1 predominated before seed imbibition but At2g18790.2 was the dominant isoform at 12 h and 48 h into dark stratification (Fig. 2f). PhyB itself plays a role in the regulation of alternative splicing [16]. Finally, polypyrimidine tract binding protein homologs (PTBs) are key regulators of alternative splicing, with 310 transcripts spliced alternatively when PTB levels are altered [37]. Notably, 28 of these 310 previously identified transcripts [37] also showed significant isoform variation in our study, suggesting that PTB mediated regulation of alternative splicing may occur for these genes during seed germination.

### The germination program includes previously unannotated loci

**Fig. 3:**
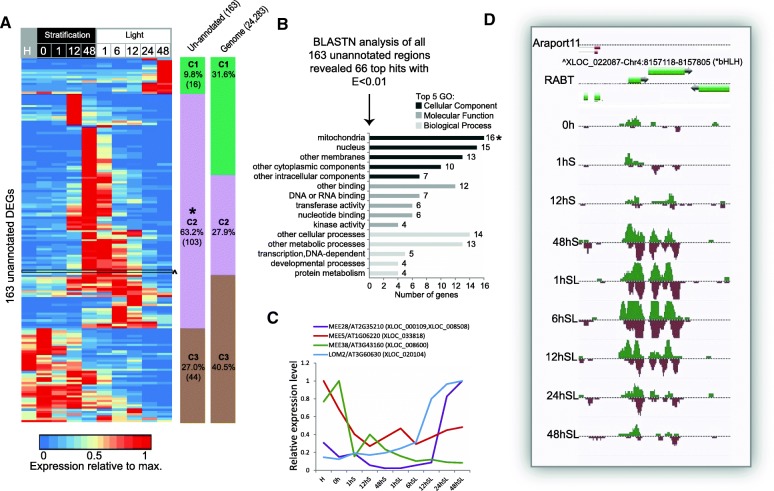
Analysis of DE unannotated loci during seed germination. **a** Relative expression levels of the 163 DE unannotated loci identified during germination. An enrichment of genes showing transient expression during germination (Cluster 2) is seen. **b** Top five enriched GO categories of the 66 genes that had significant hits (E < 0.01) following BLAST analysis. **c** Expression profiles of the four genes encoding proteins involved in developmental processes (MEE5, MEE28, MEE38 and LOM2) are shown. The identifiers of the unannotated loci that are homologous to these genes are shown in *brackets* (See Additional file 3: Table S2. for chromosomal co-ordinates). **d** Example of an unannotated locus expressed transiently during germination

The fixed set of probes used in microarrays precludes the detection of novel transcripts from unannotated loci, meaning that the current germination transcriptome datasets may be incomplete. We mined our whole transcriptome RNA-seq data to discover regions that were previously unannotated and that may be specific to germination. We generated a reference annotation based transcript (RABT) assembly from which we identified 163 unannotated differentially regulated loci (genomic coordinates are shown in Additional file 3: Table S2). These regions may represent previously unannotated whole transcripts or expressed regions of a previously undefined splice variant of a known gene. Examining the expression profiles of these regions revealed a significant enrichment (*p* < 0.05) of transiently expressed loci during germination (C2: 63.2% vs. 27.9% in the genome; Fig. 3a). This transient expression is likely the reason that these loci have not been reported previously.

To determine the potential function of unannotated loci we used the Basic Local Alignment Search Tool (BLAST) to analyse their encoding loci, identifying 66 loci that had significant alignments with annotated genes (E < 0.01). Most of the annotated genes encoded nuclear or mitochondrial proteins (nuclear-encoded); there was a significant enrichment of genes encoding mitochondrial proteins (*p* < 0.05) compared to the expected percentage based on the genome (Fig. 3b). Previous microarray studies identified a set of annotated genes showing germination-specific expression compared to other tissues, which were enriched in genes encoding nuclear and mitochondrial proteins, particularly those known to be embryo/seedling lethal [10]. Our dataset included four genes annotated as involved in developmental processes, three of which were known embryo lethal genes (maternal embryo effect [MEE]) and the fourth, a known miRNA-targeted TF involved in cell differentiation (LOM2- [44]) (Fig. 3b). These were differentially expressed during germination (Fig. 3c) and were homologous to four unannotated loci, which are consequently interesting candidates to examine for essential functions in the seed/seedling. Expression of an unannotated locus with significant alignment to another miRNA-targeted TF (At3g23690, belonging to the bHLH family) is shown in Fig. 3d. Like most of the unannotated expressed loci, this locus showed a transient peak in expression during germination before decreasing in expression in the seedling (LOC_022087; Fig. 3d). Notably, 50 of the 66 genes that were the top BLAST alignments with unannotated loci were also significantly differentially expressed during germination. However, for more than half of these, the expression of the unannotated loci did not correlate very well with the expression of the respective homologous annotated genes (27/50 had |r| < 0.5). Thus, further examination of these loci is necessary to determine whether they are unannotated exons of nearby genes or completely novel genes.

### A complex transcription factor network regulates gene expression during germination

**Fig. 4:**
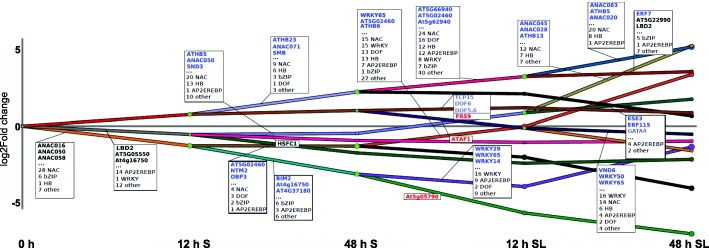
Modelling the TF network controlling germination. Simplified DREM model annotated with TFs based on DAP-seq binding data. Only the top three TFs (strongest associations) and summary of TF families involved are shown. In order to simplify the model only four time points were used to calculate the log2 fold changes of DEGs relative to 0 h: 12 h S, 48 h S, 12 h S L and 48 h S L. Transcriptionally upregulated TFs are coloured in *blue*, downregulated TFs are shown in *red*. The full model is presented in Additional file 2: Figure S3

In order to identify the key TFs driving the transcriptional dynamics during seed germination, we carried out DREM (Dynamic Regulatory Events Miner [45]) modelling of our RNA-seq time course (Fig. 4; Additional file 2: Figure S3). DREM defines transcriptional modules comprising transcripts with similar expression changes between time points. It then searches for TF-binding events enriched among the gene-encoding transcripts within modules. We subsequently hypothesise that the TFs identified may regulate those expression changes. DREM takes known TF–gene interactions as an input, which we provided from a comprehensive set of genome-wide target genes for 287 TFs, derived using published DNA affinity purification (DAP)-seq [46]. The associated TF families changed over the time course (Fig. 4). Of the two modules that are downregulated during the first 12 h of stratification, one is regulated primarily by NAC (NAM, ATAF1,2, CUC2) TFs (28 out of 43 annotated TFs) and bZIP (basic-leucine zipper) TFs (six annotated), while the other is dominated by AP2EREBP TFs (14 out of 27 annotated factors). NAC TFs are a diverse family involved in a range of developmental programs, stress and defence responses [47], but their role in germination has not been characterised so far. Our model was validated by identification of known germination-regulatory TFs. For example, ABI5 was among the bZIP TFs identified. This is a known transcriptional activator that represses germination and that is progressively downregulated over the course of germination [48, 49]. The model also identified AtHB13 as a regulatory TF during germination, which is significantly upregulated at the later stages of germination (Additional file 2: Figure S3). AtHB13 is involved in the seed to seedling transition [11], with the loss of function of AtHB13 resulting in increased primary root length.

Most bZIP TFs associated with transcriptional modules were also themselves downregulated (Fig. 4). Several modules that were upregulated from 12 h of stratification were associated with NAC and Homeobox TFs, with a large number of these TFs also being upregulated. ATHB5/6/23/33/53 appeared in multiple upregulated branches and functions in germination have been assigned to some of them: ATHB5 participates both in the ABA and gibberellin pathways [6, 50]; and ATHB23 plays roles in PhyB-dependent seed germination [51].

As the seeds were transferred to light after two days of stratification, TFs from the DOF (DNA-binding with one finger) family were inferred to play prominent roles in the upregulation of several transcription modules, which is consistent with their known roles in growth and development [52]. One example is DAG2, a positive regulator of light-mediated germination [53], which is upregulated and annotates two branches that are strongly upregulated after exposure to light (Fig. 4; Additional file 2: Figure S3). Finally, the WRKY family of TFs annotates a number of modules. WRKY TFs are involved in many different processes including germination [54], but the strongest annotations in our datasets (WRKY14/24/25/27/29/45/50/65) are not well characterised.

Although binding data are available only for a subset of Arabidopsis TFs, our model captures many of the known regulators of germination and suggests several-fold more (Fig. 4; Additional file 2: Figure S3), both expanding the role of previously described TFs to new processes and suggesting functions for as yet uncharacterised TFs. However, the strength of our approach also resides in revealing the possible combinatorial action of TFs, as factors annotated in the same branch may cooperate to activate or repress a specific set of genes. This cooperation may occur through physical interaction between TFs: DOF6 binds TCP14 [55]; although TCP14 is absent from the DAP-seq dataset, TCP15 is present, and it annotated a module that was also enriched in DOF6 and DOF5.6 targets. TCP15, DOF5.6 and DOF6 were all upregulated during germination and given the structural similarity and shared interactors between TCP14 and TCP15; TCP14 may also engage similar interactions with DOF6.

### Validation of model predictions by assessing germination rate and gene expression changes

**Fig. 5:**
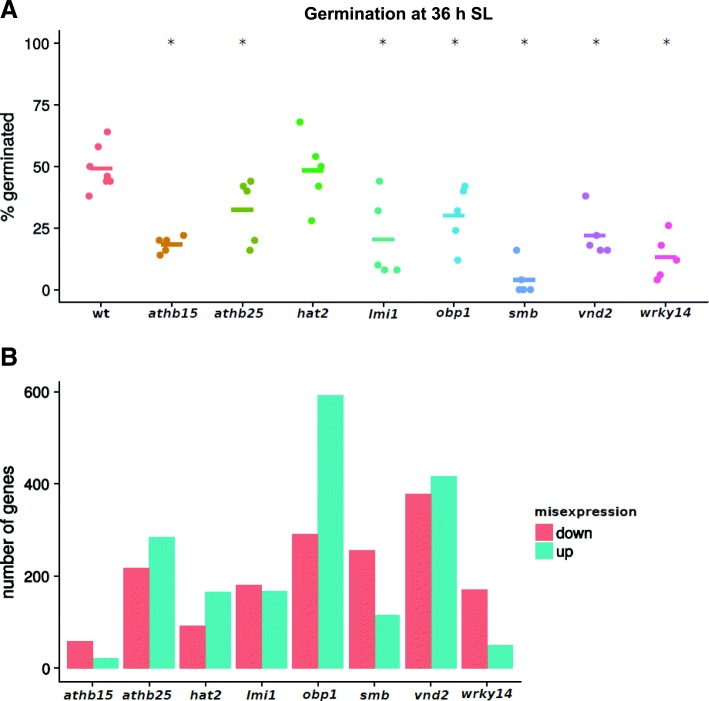
Characterisation of mutants in predicted germination regulatory TFs for model validation. **a** Percent of seeds with extruded radicles at 36 h SL. Each data point corresponds to 50 scored seeds from a single parent plant. *Horizontal bars* indicate the mean for each genotype. *Asterisks* denote significant differences compared to WT (logistic regression, *p* < 10^−5^). **b** Number of misexpressed genes at 24 h SL compared to WT (q < 0.01)

To assess the ability of our DREM model to identify novel regulators of seed germination, we procured eight homozygous knock-out lines for TFs predicted to play a role in germination: *athb15*, *athb25*, *hat2*, *lmi1*, *obp1*, *smb*, *vnd2* and *wrky14*. These TFs were selected because they were upregulated during germination and they annotated model branches upregulated after exposure to light (Additional file 2: Figure S4). All knock-out lines were confirmed to harbour a single T-DNA insertion only and the insertions were at the intended target genes. This was achieved by whole-genome resequencing, which detects T-DNA insertions (Additional file 2: Figure S5) Genotypes and homozygosity were further confirmed by polymerase chain reaction (PCR) genotyping (Additional file 3: Table S3B [56]). Seven out of eight mutant lines (all except *hat2*) germinated late, with only 5–30% of extruded radicles at 36 h in the light, compared to 50% for wild-type (WT) Col-0 (Fig. 5a). Only one TF, ATHB25, has a previously documented, indirect link with germination through an association with GA signalling and seed longevity [57]. While the remaining genes have not been shown to function in germination, OBP1 has been shown to play an important role in cell cycle regulation [58], while SMB is involved in regulating the orientation of cell division in roots [59] and ATHB15 functions in regulating stem-cell specification and organogenesis [60]. The observed phenotypes are likely caused by the T-DNA insertions we detected, but we cannot rule out deletions or translocations caused by T-DNA mutagenesis and not detected by resequencing. Thus, our DREM model is a useful tool to discover novel factors affecting germination.

We evaluated the impact of losing these TFs on gene expression by comparing the 24 h SL transcriptomes of the mutants with that of the WT (Fig. 5b; Additional file 3: Table S3A; Additional file 1: SD2). Genotypes and homozygosity of insertions were confirmed (Additional file 3: Table S3B). We found that loss of each TF caused misexpression of genes relative to the WT, but that there was no relationship between the number of misexpressed genes and the severity of the delay in germination. The *athb15* and *vnd2* mutants were similarly delayed (20% extruded radicles at 36 h SL), while 81 genes were misexpressed in *athb15* compared with 794 in *vnd2*, and 370 genes in the most delayed mutant, *smb*. Remarkably, almost all of the genes that were misexpressed in the mutants (3450 of 3453 genes compounded across the eight genotypes) were genes that underwent expression changes during germination, identified during our time series transcriptome analyses (Fig. 1; Additional file 3: Table S3A; Additional file 1: SD2). This further confirms that the TFs predicted by our model indeed participate in germination and not in widely distinct processes.

We next examined correspondence between mutations in the selected TFs and the branches of the DREM model these TFs annotated. Misexpressed genes in each mutant line were not enriched in the top DAP-seq-predicted targets of the corresponding TFs. Furthermore, only a few percent of the genes within branches of the DREM model annotated by the TFs were misexpressed in the mutants of that TF (Additional file 2: Tables S3A and S4). This reflects the fact that the transcriptional modules DREM modules represent are complex objects, regulated by a network of TFs, and therefore do not provide complete predictions of the system’s response to a constitutive mutation. Such a mutation is present throughout the lifecycle of a plant and conceivably has effects on the transcriptome more far-reaching than disruption at a specific time during germination.

### MicroRNAs may regulate gene expression during germination

**Fig. 6:**
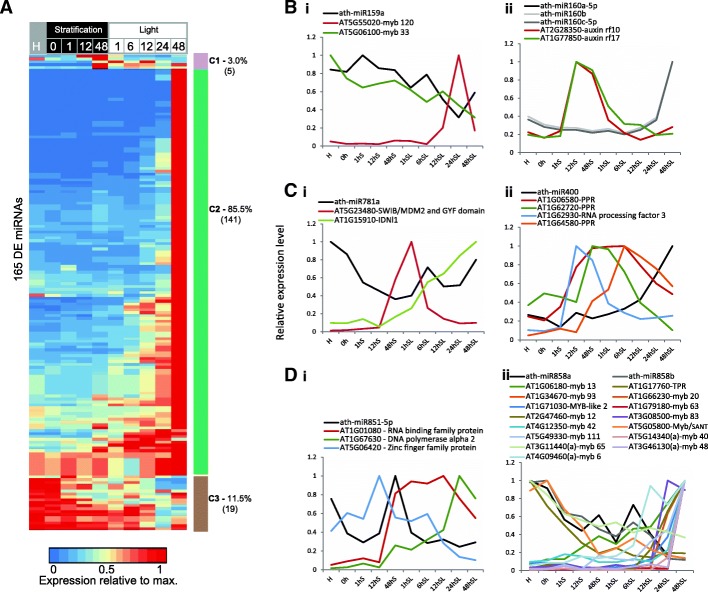
Differential expression of microRNAs over seed germination. **a** Of the annotated miRNAs, 165 were differentially expressed during germination and their relative expression levels were hierarchically clustered. **b** Expression profiles of (i) miRNA159a and (ii) miRNA160a-5p and their targets genes, which have been shown to have a role in regulation during seed germination. **c** Expression profiles of (i) miRNA781a and (ii) miRNA400 (and their target genes), which are known for a role in other (non-germination) conditions/stages in Arabidopsis. These are two of the 19 genes that showed highest expression in the dry seed. **d** Expression profiles of (i) miRNA851a and (ii) miRNA858a (and their target genes). Note that targets only predicted for miR858a are indicated with (a) next to the AGI. These are two of the five miRNAs showing a transient increased expression during germination before decreasing in abundance at the end of the time course

Small RNAs, including miRNAs and siRNAs, have regulatory roles during development in plants [4, 61]. Of previously annotated miRNAs, 165 were differentially regulated during seed germination, with the vast majority of these (85.5%) showing a significant increase in expression at 48 h SL compared to the dry seed and early hours of germination (Fig. 6a). Twenty-seven of these differentially regulated miRNAs had validated targets (miRTarBase [62], [63]), the majority of which were themselves differentially regulated, showing independent patterns of expression from the respective miRNAs, resulting in poor correlations between these (|r| < 0.5) (Additional file 3: Table S5). Most of the target genes encode proteins with DNA-binding or RNA-binding functions (Additional file 2: Figure S6). For example, miR159, miR160 and their confirmed target genes that encode MYB and auxin responsive factor TFs (Fig. 6b(i), (ii)). Both miR159 and miR160 have a functional role during seed germination via interactions with ABA [3–5]. Alterations in the levels of these miRNAs or in sensitivity of target transcripts to them altered the response of germinating seeds to ABA, which normally represses germination [3–5].

For other miRNAs, such as miR781a and miR400, their target genes are known [64] and these are differentially regulated during seed germination (Fig. 6c(i), (ii)). However, the regulatory role of these during seed germination remains to be investigated. Only five miRNAs showed a transient peak in expression during germination: ath-miR8176, ath-miR851-5p, ath-miR861-3p, ath-miR158a-5p and ath-miR779.2 (C1 in Fig. 6a). MicroRNA target prediction analysis of miR851 (Fig. 6d(i)) suggests that it may target RNA-binding, pentatricopeptide repeat (PPR) containing proteins, many of which also show a transient increase in expression during germination and have been shown to be essential for seed viability/germination [10, 65]. Nineteen miRNAs were expressed more highly in the dry seed compared to post-imbibition (C3 in Fig. 6a). These included miR159a, b and c, which are known to have a role in seed germination [4]. This indicates that closer examination of the remaining 16 miRNAs could reveal other candidates involved in regulation during germination. For example, several of the predicted targets of miR858a (Fig. 6d(ii)) were TFs that were identified as regulators of germination in our DREM model, including MYB13, MYB65 and MYB93. Thus, it is possible that miR858a has a regulatory role during germination.

### Small RNA abundance is dynamic over germination and correlates with developmental transitions

**Fig. 7:**
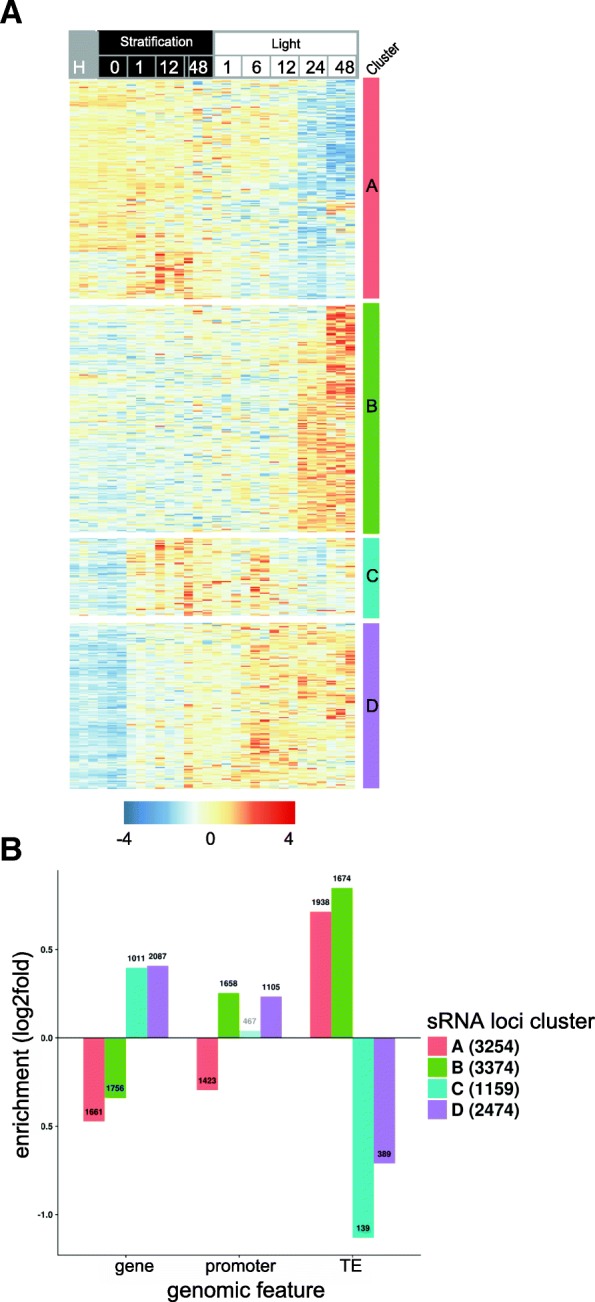
Differential expression of sRNAs during seed germination. **a** *Heatmap* of sRNA abundances for loci with differential sRNA accumulation (*p* adj < 0.01) during the time course. sRNA levels shown are the regularised log2 expression values (normalised by locus). **b** Overlap between sRNA clusters and genomic features. Non-significant enrichments are transparent. Numbers indicate the number of sRNA loci overlapping the features

During the germination time course, 10,261 sRNA loci were differentially regulated out of a total 87,054 sRNA loci identified. The analyses considered all sRNAs of 20–24 nt, including the 20–22 and 23–24 nt siRNAs. Using hierarchical clustering, the differentially regulated loci could be separated into four clusters with qualitatively distinct expression profiles (Fig. 7a). Small RNA loci from clusters A and B showed stable abundances of sRNAs until 12 h SL, after which sRNA levels starkly decreased (for cluster A) or increased (cluster B). Clusters A and B contained predominantly 23–24 nt sRNAs (77% and 74% of loci, respectively; Additional file 2: Figure S7A). The sRNAs from loci in cluster C transiently increased in abundance during stratification and until 6 h in the light, while the loci in cluster D were characterised by a progressive increase in sRNAs throughout the time course (Fig. 7a). A much smaller proportion of loci in clusters C and D contained predominantly 23–24 nt sRNAs (27% and 35% of loci, respectively; Additional file 2: Figure S7A) compared to clusters A and B. Examination of the chromosomal distribution of the sRNAs also revealed differing trends between the clusters: loci from cluster A (decreased expression in seedlings) were enriched in the centromeric heterochromatin regions, while those in cluster B (increased expression in seedlings) had a preferentially pericentromeric distribution; and loci from clusters C and D were found mostly in chromosome arms (Additional file 2: Figure S7B).

Overlapping the sRNA loci with annotated genomic features, including genes, promoters and transposable elements (TE), revealed that loci in clusters A and B were slightly enriched in TEs (60% and 50% of loci, respectively, overlapped TEs) and depleted in genes (*p* < 0.01, Fig. 7b). Given the role of 24-nt siRNAs (dominant in clusters A and B) in mediating RNA-directed DNA methylation (RdDM) and silencing of TEs [66], examination of DNA methylation patterns could give insight into this regulation during seed germination.

### Extensive DNA demethylation occurs towards the end of seed germination and in the post-germinative seedling

**Fig. 8:**
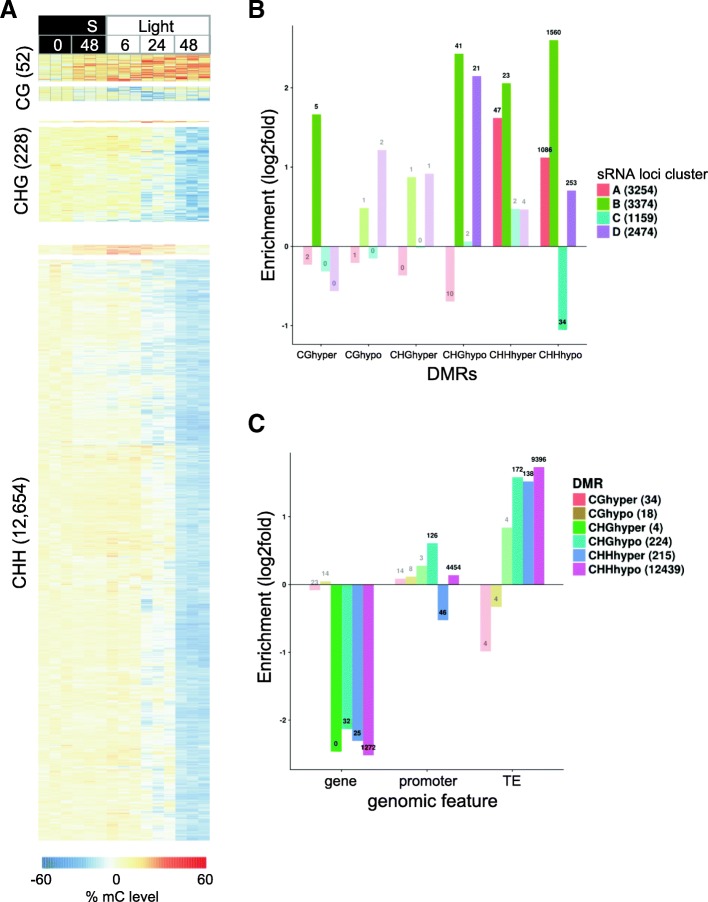
Significant demethylation occurs from seed to seedling. **a** *Heatmaps* showing DNA methylation levels (as a percentage) in DMRs in CG, CHG and CHH contexts. **b** Overlap of DMRs and sRNA loci (by cluster). **c** Overlap between DMRs and genomic features. Non-significant enrichments are transparent. Numbers indicate the number of overlaps

We investigated whether the broad transcriptional remodelling and sRNA dynamics that take place during germination were associated with epigenomic (DNA methylation) changes. The potential interaction interactions of these have not been examined previously. Analysis of DMRs in the CHH, CHG and CG contexts over the germination time course revealed very little change in the level of DNA methylation between the dry seed, after stratification (48 h S) and subsequently after 6 h exposure to light (6 h SL, Fig. 8a). However, DNA methylation levels then decreased after 24 h SL and further still after 48 h SL, by which time extensive hypomethylation was observed. Differential methylation affected 52,228 and 12,654 loci in the CG, CHG and CHH context, respectively (Fig. 8a). Overlapping DMRs in the different contexts revealed that two of 18 CG hypomethlyated DMRs overlapped the CHH hypomethylated DMRs and none overlapped CHG DMRs, whereas 216 of the 224 CHG DMRs overlapped CHH DMRs and no overlap was seen between the very few hypermethylated DMRs.

Significant overlap occurred between the DMRs and sRNA loci (by cluster) (Fig. 8b). Of the 12,439 CHH hypoDMRs, 98.8% overlapped sRNA loci that predominantly contained 23–24-nt siRNAs. The cytosine context and this overlap strongly suggested that the large decrease in DNA methylation was due to a reduced activity of the RdDM pathway, rather than the CMT pathway. However, at a majority of loci, the reduction in DNA methylation could not be attributed to a decrease in sRNA levels: only 2167 CHH hypomethylated DMRs overlapped with sRNA loci from cluster A (downregulated sRNAs), while 2189 overlapped sRNA loci from cluster B (upregulated sRNAs); and at 7684 DMRs the sRNA levels did not vary significantly. Inspecting the expression levels of the DNA methylation machinery revealed that the genes of most components were upregulated at two days in the light, including the major subunits of Pol IV and Pol V (*NRPD1* and *NRPE1*), while *DRM2* remained well expressed (Additional file 2: Figure S8). Although the expression of demethylases *DME* and *DML2* also increased, the expression of the major demethylase *ROS1* was much lower than in the dry seeds (Additional file 2: Figure S8). Furthermore, the coincidence of DNA demethylation with the onset of DNA replication in cells of the seed [67] argues for a mechanism of passive demethylation rather than active demethylation. Comparing the methylation levels of the DMRs with publicly available methylomes of *A. thaliana* embryo, endosperm and leaves revealed that the demethylated seedling at 48 h SL most closely resembles the leaf methylome (Additional file 2: Figure S9).

To assess whether the failure to maintain high levels of DNA methylation may be due to the protection of the DNA by TFs, we quantified the overlap of DMRs with the known binding sites of the TFs from the families that dominated the end of the time course (based upon the DREM model). Overall, 3150 CHH DMRs overlapped TF binding sites, only slightly less than expected by chance (Additional file 3: Table S6). However, the different families of TFs displayed great disparities in their overlaps with DMRs: the binding sites of AP2EREBP factors were strongly depleted in DMRs (32-fold compared to chance), which may be due in part to their binding motifs containing a CCG/CGG motif [46] (and thus constituting MET1/CMT3 targets rather than RdDM sites). Conversely, the binding sites of DOF and HB TFs were slightly enriched in DMRs (1.3-fold and 1.5-fold, respectively, corresponding to 782 and 1330 DMRs). Therefore, three-quarters of the CHH DMRs did not overlap binding sites from this subset of TFs, which would suggest that protection by TFs does not play a major role in the passive loss of DNA methylation at the seed-seedling transition stage. However, the binding data are not comprehensive and other TFs may bind more DMRs, so the full extent of the contribution of TF binding to loss of DNA methylation remains to be determined.

We next asked whether the decrease in CHH methylation may be linked to the transcriptional reprogramming of the emerging seedling. A total of 9541 and 7242 genes were upregulated and downregulated, respectively, between the 12 h SL and 48 h SL time points. In total, 1053 and 799 had CHH hypoDMRs in their promoters (from 1 kb upstream to 200 bp downstream of the transcriptional start site). Though there was no strong bias for upregulation of genes with hypomethylated promoters, and overall the DMRs were only very slightly enriched in promoter regions (Fig. 8c), hypomethylation of the promoters of more than 1800 DEGs is considerable.

## Discussion

In this study, we have characterised the regulatory network of transcriptomic and epigenomic changes that control Arabidopsis seed germination. Extensive transcriptome remodelling occurred from the dry seed stage, through stratification, germination and post-germination, following exposure to light. This included hundreds of previously unannotated loci, which are possibly germination-specific. We found that alternative splicing and divergent isoform usage was common in the germination transcriptome. TF families with direct regulatory roles governing specific transcriptional outputs were identified using time-series modelling. Substantial changes in sRNA populations also occurred over our time course. These included miRNAs, most of whose target transcripts were also differentially regulated across germination. However, the largest changes were in the abundance of 23–24-nt siRNAs, which are associated with RdDM loci. This correlated with genome-wide DNA hypomethylation, predominantly in the CHH context, as the seed epigenome transitioned from an embryo-like state to a vegetative seedling state. We note that extensive changes in DNA methylation, transcripts and sRNA abundance occurred during stratification, despite the lack of dormancy in the seeds we examined [40, 41]. These changes likely reflect perception of both low temperature and imbibition by the seed, though it should be highlighted that Col-0 accession seeds harvested from plants grown at 22 °C do not exhibit secondary dormancy induced by chilling [68]. Our analyses provide unprecedented detail of the germination process that will be useful in future seed optimisation efforts.

We identified 163 unannotated differentially regulated loci (Fig. 3). These predominantly showed a transient expression pattern during germination and were homologous with genes encoding mitochondrial proteins. This is consistent with the resumption of active metabolism that characterises this phase of germination [69]. A number of mutations in related transiently expressed genes are embryo-lethal [10, 69]. Consequently, the unannotated loci we identified warrant further investigation of whether they are essential for successful germination.

One role of alternative splicing during germination is to diversify the transcriptome [14, 37, 70, 71]. This is achieved in part by divergent isoform usage of transcripts encoding the alternative splicing machinery themselves (Fig. 2). Differential regulation of the splicing machinery has growth and developmental effects in petals and roots [38]. Some of these same components were alternatively spliced during germination, suggesting they may also play a role in seed germination (Fig. 2).

Alternative splicing also influenced light signalling genes during germination, in agreement with a prior study (Fig. 2) [14]. Connections exist between alternative splicing, light signalling and energy availability during early photomorphogenesis [14]. Among the 620 genes with divergent isoform usage during germination (Fig. 2), 16 were annotated to take part in responses to light. These included *PhyB* (At2g18790), *Photosystem II Light Harvesting Complex Gene 2.1* (*LHCB2.1*; AT2G05100), *MYB65* (At3g11440) and *PIF6* (At3g62090). Alternative splicing of *PIF6* results in altered rates of ABA-dependent seed germination [37], while PhyB is required for promoting seed germination in the light via GA signalling [70, 71]. Light perception, involving PhyB, plays an important role in the spatial rearrangement of the nucleus, specifically chromatin decondensation in the later stages of seedling growth, during the transition to photomorphogenesis [72]. This is likely important for post-embryonic transcriptional reprogramming, as the seed genome becomes more active and a greater number of genes are expressed. In combination, this confirms that alternative splicing enables integration of light responses to the regulatory network controlling seed germination.

In our study, we illustrate the complexity of TF interactions during germination using DREM modelling of the time-series transcriptomes (Fig. 4). Numerous members of different TF families are involved at each developmental transition. Previous studies have focused on single or small numbers of TFs from specific families, but here we reveal that the regulatory network is much richer. Our modelling used target gene data obtained from an in vitro approach [46]. This method was validated as providing accurate data of true in vivo target genes, but it is likely that TF behaviour and expression varies between growth stages and tissues. Obtaining in planta binding data from seeds or individual seed tissues will reveal in greater detail the target genes that are effectively directly bound during germination. In accordance with published germination studies, we identified ABI5, ATHB23 and DAG2 among the known regulators of germination, with the latter two involved in PhyB-dependent seed germination [51, 53].

In addition to identifying known regulators of germination, our model also predicts many novel germination-regulatory TFs, whose roles have either not been characterised or have not been linked to germination. Using homozygous T-DNA insertion lines, we show that impairments of predicted germination-regulatory TFs cause delays in germination for seven out of eight candidates (*athb15*, *athb25*, *lmi1*, *obp1*, *smb*, *vnd2* and *wrky14*). The phenotypes observed were likely caused by the T-DNA insertions. However, the resequencing approach we used does not rule out the possibility that translocations or deletions influencing phenotype may also exist in these lines. In-depth characterisation of these TFs and genes misexpressed in their mutants will allow the reconstitution of a more comprehensive regulatory network governing germination, while application of inducible systems that combine multiple TFs may allow finer control over transcriptional modules. This modelling approach notably expands the number of validated germination-regulatory TFs and increases our understanding of the regulatory language that controls gene expression during germination.

The populations of miRNAs and siRNAs both changed over germination, indicating that these two distinct branches of the RNA-silencing mechanism are involved in seed–seedling transitions (Figs. 6 and 7). Previous studies suggest that alternative splicing regulates miRNA biogenesis [17]. This presents an interesting prospect for future investigation, given the extent of alternative splicing we and others observe during germination and early photomorphogenesis [14]. MicroRNAs are also known to mediate changes in de novo DNA methylation in Arabidopsis by targeting the genes involved in DNA methylation [63], including miRNA781a and miRNA773a, which target *INVOLVED IN DE NOVO 2 - like 1* and *DRM2* [63]. Both of these miRNAs and their confirmed targets were differentially regulated during seed germination and may have contributed to the extensive DNA hypomethylation we observed (Figs. 6 and 8).

A large number of differentially regulated 20–22-nt siRNA loci overlapped differentially regulated genes (Additional file 2: Figure S7C). This size class is predominantly involved in post-transcriptional regulation of transcripts by targeted degradation [73]. A simple model of post-transcriptional gene silencing would predict negative correlations between abundances of mRNAs and their targeting siRNAs. However, as we observed both positive and negative correlations between these, more complex regulatory systems such as negative feedback loops may be involved (Additional file 2: Figure S7C). Consequently, the role of siRNAs in controlling gene expression during germination cannot be precisely determined. Studying germination in mutant plants deficient in siRNA pathways will shed more light on this mechanism.

Extensive DNA demethylation occurred between the seed and post-germinative seedling, corresponding with the onset of DNA replication and cell division (Fig. 8). This decrease in methylation occurred almost exclusively in the CHH context and at RdDM loci. Rather than a complete removal of methylation, most DMRs underwent a quantitative reduction in DNA methylation. At most DMRs, no changes in sRNA abundance were recorded, although about 10% of DMRs could be associated with decreases or increases in sRNAs. Combined with the high expression of the RdDM machinery at this stage, these results suggest that the decrease of methylation in the seedling is mostly passive and due to a lesser efficiency of the RdDM machinery compared to the period of seed development. Indeed, comparison of the methylation profiles of our time points with publicly available datasets for embryo, endosperm and three-week-old leaf samples demonstrate that the hypermethylated seed up to 12 h SL is most similar to the embryo samples, whereas the emerging seedling adopts a profile that closely resembles one that is observed in leaves (Additional file 2: Figure S9). This is consistent with what is known about the epigenetic reprogramming of the zygote, with increased RdDM activity supported by companion-cell-derived sRNAs [25, 61]. More than 1800 demethylated regions correspond to promoters that change in activity during the seed–seedling transition. Although the upregulation of genes through promoter DNA hypomethylation does not appear to be a major mechanism of gene regulation during germination, it may play a role for a specific set of genes. Alternatively, changes in promoter activity may impair the recruitment of the RdDM machinery and thus contribute to the loss of methylation at these sites.

DNA methylation affects the ability of many TFs to bind DNA [46]. The majority of TFs identified as potential germination regulators by our DREM model are negatively affected by DNA methylation [46]. Consequently, the DNA demethylation taking place in the emerging seedling may generate new TF binding sites, thereby re-shaping the regulatory network that governs vegetative growth. This is supported by the observation that the promoter of a TF involved in germination under salt stress, MYB74, loses DNA methylation during salt stress and that this results in increased expression of the *MYB74* gene [74]. *MYB74* over-expressing plants also displayed hypersensitivity to salt during seed germination [74]. During germination in our study, MYB74 shows significant induction in the 48-h seedling (48 h SL), while a region in its promoter targeted by siRNAs exhibited decreased methylation (Additional file 2: Figure S10).

## Conclusions

We present a deep characterisation of the dynamic network of interactions between transcription, alternative splicing, sRNAs and DNA methylation during germination. We reveal extensive and stage-specific switches in isoform usage, a layer of complexity in the germination transcriptome that was previously unknown. Modelling regulatory events along the germination time course allowed us to organise known TFs controlling germination and to predict a large number of novel regulatory TFs. We validated that several of the predicted regulatory TFs altered germination rate and gene expression during germination, confirming the value of our model. Finally, we uncovered the genome-wide demethylation undergone by the germinating seedling at the onset of cell division. This study provides a deeper and more comprehensive understanding of seed germination, which will potentially contribute to trait optimisation efforts.

## Methods

### Arabidopsis growth and tissue collection

For the time course of germination (Col-0 only; Fig. 1a(i)), Arabidopsis plants were grown to maturity at 22 °C under long-day conditions and seeds were collected (freshly harvested – H) from this single batch of plants. The seeds were then kept for two weeks under dry, dark conditions to ripen before being collected (for 0 h samples) and then plated onto MS media plates (containing 3% sucrose). Samples were collected after 1 h of cold (4 °C) dark stratification (S), 12 h S and 48 h S before being transferred to continuous light (SL) (at 22 °C) and collected after 1 h (SL), 6 h SL, 12 h SL, 24 h SL and 48 h SL. Three biological replicates were collected. See [10] for details.

For the validation of DREM predictions, we procured eight homozygous knock-out lines of TFs predicted to play a role in germination, available in the CS27941 set; [75]. WT Col-0, athb15 (AT1G52150, SALK_140350C), athb25 (AT5G65410, SALK_133857C), *hat2* (AT5G47370, SALK_091887C), *lmi1* (AT5G03790, SALK_131946C), *obp1* (AT3G50410, SALK_049540C), *smb* (AT1G79580, SALK_143526C), *vnd2* (AT4G36160, SALK_026864C) and *wrky14* (AT1G30650, SALK_105170C) were all grown in parallel to maturity at 22 °C under long-day conditions and seeds were collected from individual plants. After five days of drying in the dark, the seeds were plated onto MS media plates (3% sucrose) as before and underwent 48 h of stratification at 4 °C in the dark before being transferred to a growth cabinet (22 °C, constant light at 100 μmol photons.m^−2^.s^−1^). For germination scoring, 50 seeds from at least five individual plants per genotype were monitored for radicle extrusion at 26, 36 and 49 h SL. For RNA-seq, seeds pooled from multiple parent plants were collected at 24 h SL in duplicates.

### Validation of the mutant lines by whole-genome resequencing

In addition to genotyping the eight homozygous mutant lines that we assessed for germination kinetics by regular PCR with primers designed on T-DNA express (http://signal.salk.edu/cgi-bin/tdnaexpress and Additional file 3: Table S3B) [56, 75], we performed whole-genome resequencing to ensure that there were single insertions only and that these were in the intended target genes. DNA was extracted from two-week-old mutant and WT seedlings with the QIAgen DNeasy extraction kit and genomic libraries were constructed with the Nextera DNA Library Preparation kit (Illumina) according to the manufacturer’s instructions. Libraries were enriched for large inserts by size selection with a 0.5X SPRI beads clean up. Sequencing in paired-end mode (75 bp) on an Illumina NextSeq 500 yielded 9–30 M reads per library. Reads were trimmed with trimgalore (v0.4.2) with options --phred33 --paired --nextera and mapped with bowtie2 (2.2.29) (CITE Langmead 2012) with option -X 1500 onto the TAIR10 genome with the pROK2 T-DNA sequence as a supplementary chromosome [76, 77]. Read pairs with one read mapping to the T-DNA and its mate mapping to the genome were extracted from the SAM output with awk ‘($3 == “pROK2_T-DNA_only” && $7 ! = “=”)’, to identify T-DNA insertion sites. Each mutant line had only one T-DNA insertion, at the predicted position, supported by at least 48 pairs, while no such pairs were found in WT Col-0 (Additional file 2: Figure S5).

### RNA isolation and RNA-seq

For the samples collected from the time course of germination (Col-0 only; Fig. 1a(i)), the Ambion Plant RNA isolation aid and RNAqueous RNA isolation kit were used for RNA isolation. RNA quality and integrity were determined using the Nanodrop 1000 Spectrophotometer and Agilent Bioanalyser. Only high-quality RNA samples (Abs260/280 nm ratios of 2.0–2.1) were used for RNA-seq library generation with the Illumina TruSeq Total RNA sample prep kit. RNA-seq libraries were multiplexed and loaded per lane into the Illumina HiSeq flow cell v3. All sequencing protocols were carried out as per the manufacter’s instructions using the Illumina HiSeq 1000 and HiSeq control software.

For the samples collected from the eight TF mutant lines and Col-0 in parallel (validation of the DREM predictions), RNA from the 24 h SL timepoint was extracted with the Spectrum RNA extraction kit (Sigma) in duplicates for each genotype. RNA-seq libraries were prepared with the Illumina TruSeq mRNA kit, pooled and sequenced on one NextSeq500 flow cell.

### Gene-level differential expression

RNA-seq reads were trimmed with trimgalore v0.4.2 and mapped onto the Arabidopsis TAIR10 genome with the Araport11 transcriptome annotation using HISAT2 v2.0.5 [78]. Gene counts were extracted with featureCounts v1.5.1 [79] and analysed with DESeq2 v1.10.1 [80]. Genes were considered differentially expressed during the time course when the adjusted *p* value of the likelihood ratio test (reduced model, no effect of time vs. full model, time factor with ten levels) was < 0.01. Expression levels of the DE loci were classified into clusters by hierarchical clustering based on Euclidean distance. Functional enrichment analysis was carried out using the GO Enrichment Analysis tool (http://geneontology.org/).

### Isoform quantification

Alternative splice variants were quantified with Salmon v0.7.2 [81]. Araport11 transcripts were quasi-indexed with k = 31 and RNA-seq libraries were quantified with 30 bootstraps and the VB optimizer. Transcript quantifications were then analysed for differential accumulation using Sleuth v0.28.1 [82], using a likelihood ratio test between the reduced and full model as before.

### Identification of unannotated loci

The cufflinks package [83], version 2.2.0 (Bowtie2 v 2.2.0-beta7 and Tophat v2.2.0), was used with the TAIR10 genome sequence to align the RNA-seq reads to the genome using the gene model annotation file (GFF) from TAIR10 with the following options: −b2-sensitive –r 0 –mate-std-dev 100 –g 1 –G. The RABT assembly was generated using the resulting aligned reads with Cufflinks v 2.2.0 in discovery mode, in order to identify previously unannotated genes (as previously described [83]. To do this, reads corresponding to the dry seed (0 h), 48 h S and 48 h SL were merged using Cuffmerge and the TAIR10 assembly as a reference to create the RABT assembly for transcript abundance quantification. In this way, 881 un-annotated regions (>200 bp) were identified and 394 of these were differentially regulated. Of these, 231 regions (~60%) overlapped with genes annotated in Araport11, supporting this method of identifying previously unannotated loci and leaving 163 previously unannotated differentially regulated loci.

### Modelling the regulatory network

Log2-fold changes relative to 0 h for the DEGs at 12 h S, 48 h S, 12 h SL and 48 h SL were used to annotate the transcriptional dynamics with likely regulatory TFs using the DREM framework [45]. To decrease the size of the TF–gene interaction dataset [46], we kept the strongest 25% peaks for each of the 287 TFs and identified their presumed target with ChIPpeakAnno [84]. Only associations with a *p* value < 10^−7^ are shown. TF binding data, gene expression data, parameters and output model used in DREM modelling are shown in Additional file 1: SD3.

### Small RNA-seq protocol

For sRNA analysis, 1 μg of total RNA was used for library preparation with the Small RNA sample preparation kit (Illumina). Three biological replicates were conducted per time point. Briefly, Illumina RNA adapters were sequentially ligated to the small RNA molecules. These adapter-ligated samples were reverse-transcribed and PCR-amplified before gel purification for size selection (15–30-nt inserts) [30]. The libraries were multiplexed and sequenced for 96 cycles using the Illumina HiSeq 1000, as per the manufacturer’s instructions.

### Small RNA analysis

Small RNA-seq reads were trimmed with trimgalore (v0.4.2) and sequences of 18–24 nt in length were mapped and clustered onto the Arabidopsis TAIR10 genome with ShortStack v3.6 [85]. The read counts on all 87,000 defined clusters were subjected to differential analysis with DESeq2 v1.10.1 [80]. Small RNA loci were considered to vary during the time course when the adjusted p value of the likelihood ratio test (reduced model, no effect of time vs. full model, time factor with ten levels) was < 0.01. Differential loci were classified into four clusters by hierarchical clustering based on the Euclidean distance of the regularised logarithm of counts.

Counts for miRNAs annotated in Araport11 were obtained with featureCounts from the sRNA libraries mapped onto TAIR10 with bowtie v1.1.2 [86] with options -v 1 -a --best --strata. For visualisation in JBrowse we used the sRNA plugin (https://github.com/bhofmei/jbplugin-smallrna), courtesy of Brigitte Hofmeister.

### Genomic DNA extraction and MethylC-seq

Genomic (g)DNA was extracted from the seeds/seedlings using the Qiagen DNeasy Plant mini kit. Purified gDNA (600 ng) was used for MethylC-seq library preparation after spiking in 0.5% lambda DNA (N6-methyladenine-free) (New England BioLabs) [87]. Three biological replicates were conducted per time point. Following bisulfite conversion and purification, the adapter-ligated DNA molecules were sequenced using the Illumina HiSeq 1000, following manufacturers’ instructions. For visualisation in JBrowse we used the methylation plugin (https://github.com/bhofmei/jbplugin-methylation), courtesy of Brigitte Hofmeister. Note for Fig. 1b and Additional file 2: Figure S10, data were shown within the AnnoJ genome browser (http://www.annoj.org/).

### DMR detection

DMRs were identified using HOME (v0.1) (https://github.com/Akanksha2511/HOME). Briefly, single-end MethylC-seq reads were trimmed with trimgalore (v0.4.2) and mapped to TAIR10 genome with Bismark v0.16.3 [88] and default parameters. Deduplicated reads (deduplicate_bismark) were used to generate genome-wide cytosine reports (bismark_methylation_extractor, bismark2bedGraph, coverage2cytosine). The processed data were then used to identify timeseries DMRs for the three contexts (CG, CHG and CHH) using HOME-timeseries with default parameters. We further filtered out regions whose maximum absolute difference in methylation during the time course was lower than 20%. Hierarchical clustering of the methylation differences relative to the first time-point (0 h) distinguished hypermethylated and hypomethylated regions.

### DMR analysis

Overlaps between DMRs, differential sRNA loci and genomic features were computed with the Genome Association Tester (GAT) v1.0 and default parameters. Bootstraps of overlaps were generated on the whole TAIR10 genome.

## Additional files

Additional file 1: SD1: List of genes that are differentially expressed during the germination time course. SD2: Lists of genes that are misexpressed in each of the mutants at 24 h SL. SD3: TF binding data, gene expression data, parameters and output model used in DREM modelling. (ZIP 2580 kb)

Additional file 2: Figure S1. Transcriptomic responses over seed germination. **Figure S2.** Correlation between the main two isoforms of genes with multiple variants during germination. **Figure S3.** Modelling the TF network controlling germination. **Figure S4.** Reduced DREM model showing the TFs selected for validation. **Figure S5.** Results of the whole-genome resequencing of the eight mutant lines of DREM-predicted TFs. **Figure S6.** Differential expression of miRNAs over seed germination. **Figure S7.** sRNA size classes, distribution along the five chromosomes and correlation between sRNA loci and expression of their target genes. **Figure S8.** Expression of the methylases, demethylases and Pol IV/V main subunits. **Figure S9.** Hierarchical clustering of methylomes from our time course and other Arabidopsis tissues, based on the Euclidean distance of methylation levels of the 12,654 CHH DMRs, agglomerated by complete linkage. **Figure S10.** AtMYB74 during seed germination. (PDF 9278 kb)

Additional file 3: Table S1. The 620 genes showing isoform variation during seed germination. **Table S2.** The 163 DE unannotated loci identified during seed germination. **Table S3A.** Number of misexpressed genes at 24 h SL in each genotype and subsets that are differentially expressed during germination and DAP-seq-predicted targets of the corresponding TF. **Table S3B.** Primers used for genotyping the mutant lines. **Table S4.** Number of misregulated genes in each branch of the DREM model (Fig. S) at 24 h SL in each mutant. **Table S5.** Correlation between the expression profiles of miRNAs and their respective confirmed target gene(s) for all those shown in Figure S6. **Table S6.** Overlap between CHH DMRs and TF binding sites. (XLSX 52 kb)

## Abbreviations

ABA: Abscisic acid
AGI: Arabidopsis Gene Identifier
DAP-seq: DNA affinity purification sequencing
DB: Database
DEG: Differentially expressed gene(s)
DMR: Differentially methylated region
GA: Gibberellic acid
gDNA: Genomic DNA
GO: Gene Ontology
MEE: Maternal embryo effect
miRNA: microRNA
PPR: Pentatricopeptide repeat
S: Stratified
siRNA: Small interfering RNA
SL: (exposed to) Light (after 48 h stratification)
sRNA: Small RNA
TAIR: The Arabidopsis Information Resource
TE: Transposable element
TF: Transcription factor
