# Supplementary material for: Extensive transcriptomic and epigenomic remodelling occurs during Arabidopsis thaliana germination
Source: Genome Biol. 2017 Sep 15;18:172. doi: 10.1186/s13059-017-1302-3 (PMC5599894; doi:10.1186/s13059-017-1302-3)
Supplement: Supplementary file 2 — Transcriptomic responses over seed germination. Figure S2. Correlation between the main two isoforms of genes with multiple variants during germination. Figure S3. Modelling the TF network controlling germination. Figure S4. Reduced DREM model showing the TFs selected for validation. Figure S5. Results of the whole-genome resequencing of the eight mutant lines of DREM-predicted TFs. Figure S6. Differential expression of miRNAs over seed germination. Figure S7. sRNA size classes, distribution along the five chromosomes and correlation between sRNA loci and expression of their target genes. Figure S8. Expression of the methylases, demethylases and Pol IV/V main subunits. Figure S9. Hierarchical clustering of methylomes from our time course and other Arabidopsis tissues, based on the Euclidean distance of methylation levels of the 12,654 CHH DMRs, agglomerated by complete linkage. Figure S10. AtMYB74 during seed germination. (PDF 9278 kb) [file 13059_2017_1302_MOESM2_ESM.pdf]

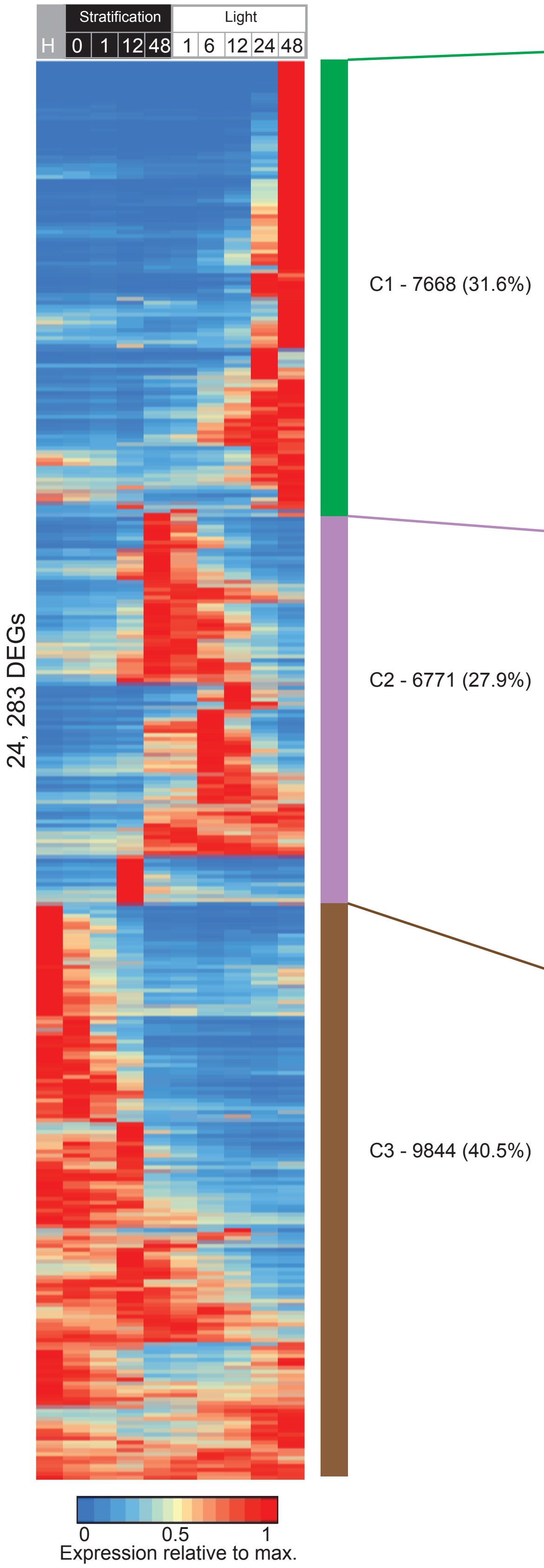

GO Biological process	FE	p-val
hydrogen peroxide catabolic process (GO:0042744)	3	3.00E-09
plant-type secondary cell wall biogenesis (GO:0009834)	2.9	3.06E-03
hydrogen peroxide metabolic process (GO:0042743)	2.8	2.49E-08
pectin biosynthetic process (GO:0045489)	2.7	8.32E-03
chlorophyll biosynthetic process (GO:0015995)	2.7	2.75E-02
root hair elongation (GO:0048767)	2.6	3.61E-02
root hair cell development (GO:0080147)	2.5	4.68E-03
trichoblast differentiation (GO:0010054)	2.5	2.87E-04
root hair cell differentiation (GO:0048765)	2.4	9.17E-04
trichoblast maturation (GO:0048764)	2.4	9.17E-04
cell maturation (GO:0048469)	2.4	1.36E-03
reactive oxygen species metabolic process (GO:0072593)	2.4	1.62E-06
root epidermal cell differentiation (GO:0010053)	2.3	3.61E-04
cell wall polysaccharide metabolic process (GO:0010383)	2.3	7.73E-05
transmembrane receptor protein tyrosine kinase signaling pathway (GO:0007169)	2.2	5.06E-05
hemicellulose metabolic process (GO:0010410)	2.2	9.61E-04
enzyme linked receptor protein signaling pathway (GO:0007167)	2.2	7.12E-05
lignin metabolic process (GO:0009808)	2.2	7.37E-03
plant-type cell wall organization (GO:0009664)	2.2	9.95E-05
phenylpropanoid metabolic process (GO:0009698)	2.2	2.21E-06
cell wall macromolecule metabolic process (GO:0044036)	2.2	1.64E-05
plant epidermal cell differentiation (GO:0090627)	2.2	4.13E-04
polysaccharide biosynthetic process (GO:0000271)	2.1	2.69E-07
fatty acid biosynthetic process (GO:0006633)	2.1	2.29E-06
pigment biosynthetic process (GO:0046148)	2.1	1.05E-03

GO Biological process	FE	p-val
ribosomal large subunit biogenesis (GO:0042273)	3.5	4.84E-02
ribosomal small subunit biogenesis (GO:0042274)	3.4	1.48E-04
ribosome assembly (GO:0042255)	3.4	3.14E-04
protein targeting to mitochondrion (GO:0006626)	3.3	8.21E-03
protein localization to mitochondrion (GO:0070585)	3.3	8.21E-03
establishment of protein localization to mitochondrion (GO:0072655)	3.3	8.21E-03
ribosome biogenesis (GO:0042254)	3.2	7.45E-27
translation (GO:0006412)	2.9	1.05E-55
peptide biosynthetic process (GO:0043043)	2.9	5.76E-56
ribonucleoprotein complex biogenesis (GO:0022613)	2.9	7.31E-27
amide biosynthetic process (GO:0043604)	2.8	1.29E-54
peptide metabolic process (GO:0006518)	2.7	3.29E-52
translational elongation (GO:0006414)	2.6	1.01E-03
cellular amide metabolic process (GO:0043603)	2.5	9.01E-49
ribonucleoprotein complex assembly (GO:0022618)	2.5	2.25E-04
ribonucleoprotein complex subunit organization (GO:0071826)	2.5	2.25E-04
rRNA metabolic process (GO:0016072)	2.4	2.62E-03
rRNA processing (GO:0006364)	2.3	6.52E-03
ncRNA processing (GO:0034470)	2.3	5.59E-06
ncRNA metabolic process (GO:0034660)	2.1	2.32E-06
organonitrogen compound biosynthetic process (GO:1901566)	2	8.32E-35
RNA processing (GO:0006396)	1.7	1.45E-05
organonitrogen compound metabolic process (GO:1901564)	1.6	5.01E-22
cellular macromolecular complex assembly (GO:0034622)	1.6	1.77E-02
gene expression (GO:0010467)	1.6	1.54E-38

GO Biological process	FE	p-val
RNA splicing (GO:0008380)	2	1.55E-06
histone modification (GO:0016570)	1.9	1.03E-02
mRNA processing (GO:0006397)	1.9	2.90E-07
covalent chromatin modification (GO:0016569)	1.8	4.03E-05
mRNA metabolic process (GO:0016071)	1.8	1.96E-07
regulation of gene expression, epigenetic (GO:0040029)	1.7	4.89E-02
chromatin organization (GO:0006325)	1.7	1.86E-06
small molecule catabolic process (GO:0044282)	1.7	3.77E-02
ubiquitin-dependent protein catabolic process (GO:0006511)	1.7	1.96E-06
modification-dependent protein catabolic process (GO:0019941)	1.7	2.16E-06
modification-dependent macromolecule catabolic process (GO:0043632)	1.7	2.71E-06
regulation of post-embryonic development (GO:0048580)	1.6	1.74E-04
protein catabolic process (GO:0030163)	1.6	1.42E-06
cellular protein catabolic process (GO:0044257)	1.6	3.32E-06
proteolysis involved in cellular protein catabolic process (GO:0051603)	1.6	9.55E-06
regulation of reproductive process (GO:2000241)	1.6	7.47E-03
RNA processing (GO:0006396)	1.6	2.04E-07
cellular macromolecule catabolic process (GO:0044265)	1.6	2.04E-07
protein modification by small protein conjugation or removal (GO:0070647)	1.6	6.20E-09
cellular protein localization (GO:0034613)	1.6	8.41E-05
protein ubiquitination (GO:0016567)	1.6	6.43E-07
protein localization (GO:0008104)	1.6	8.71E-09
regulation of cellular component organization (GO:0051128)	1.6	2.04E-02
protein modification by small protein conjugation (GO:0032446)	1.5	5.23E-07
vesicle-mediated transport (GO:0016192)	1.5	1.10E-03

Fig. S1 Transcriptomic responses over seed germination. The 24,283 differentially expressed genes (24,120 from Araport11 and 163 newly identified unannotated transcripts) were hierarchically clustered and GO Enrichment (Biological process) was carried out for the genes in each of the clusters. The top 25 significantly over-represented ($p < 0.01$) GO terms based on fold-enrichment (FE) are shown as well as the associated p-values.

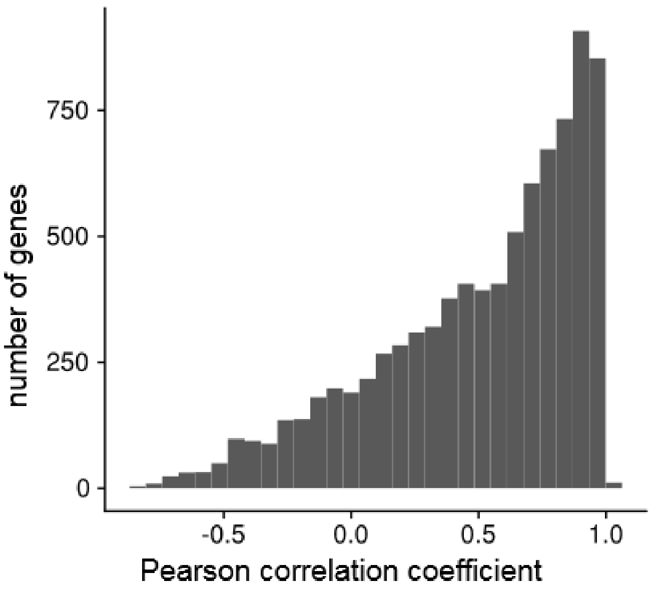

Fig. S2 Correlation between the main two isoforms of genes with multiple variants during germination.

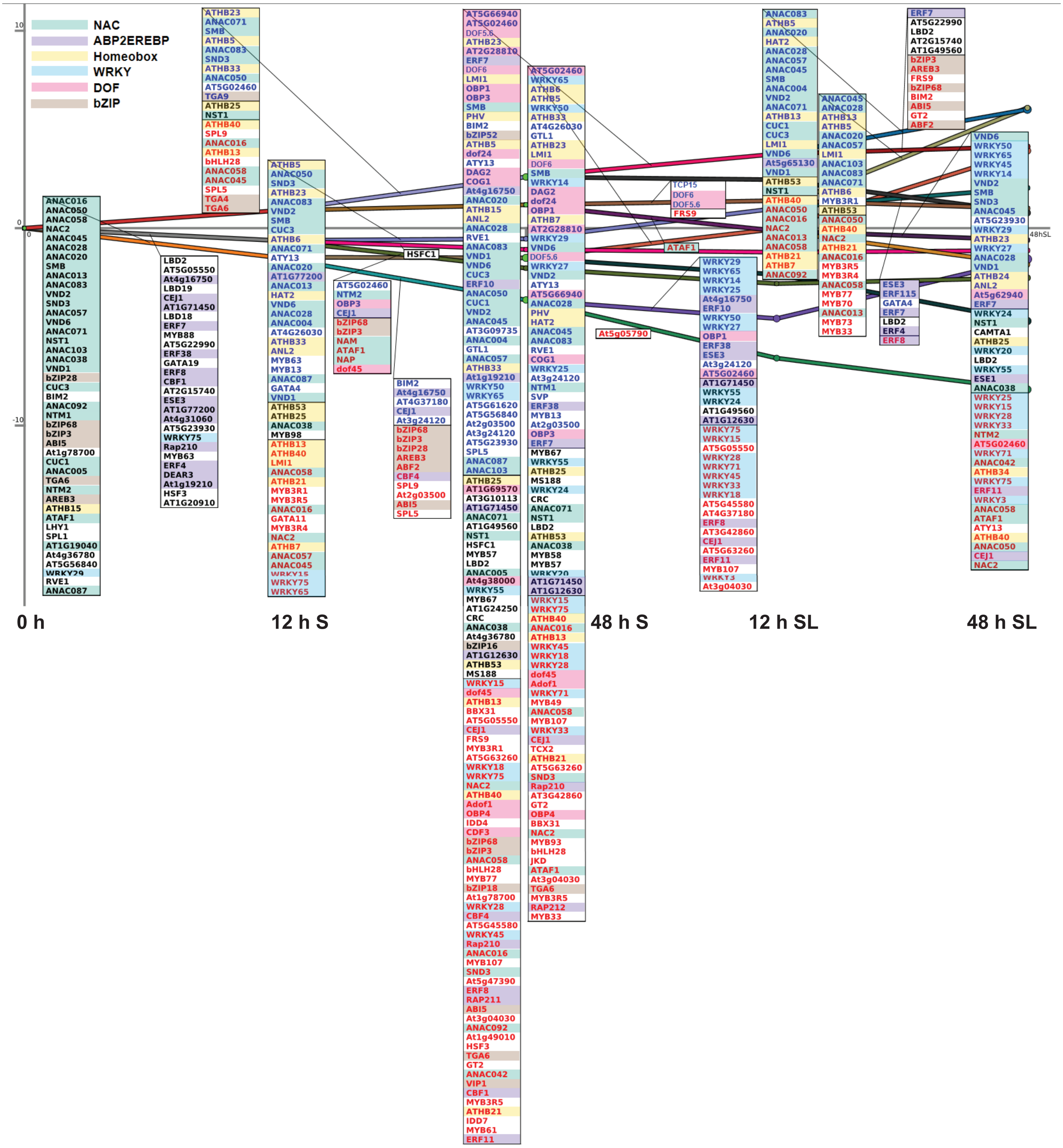

Fig. S3 Modelling the TF network controlling germination. DREM model annotated with TFs based on DAP-seq binding data. In order to simplify the model only four time points were used to calculate the log2 fold changes of DEGs relative to 0 h: 12 h S, 48 h S, 12 h SL and 48 h SL. Transcriptionally up-regulated TFs are coloured in blue, transcriptionally down-regulated TFs are shown in red. TFs belonging to major families are highlighted in the colours indicated.

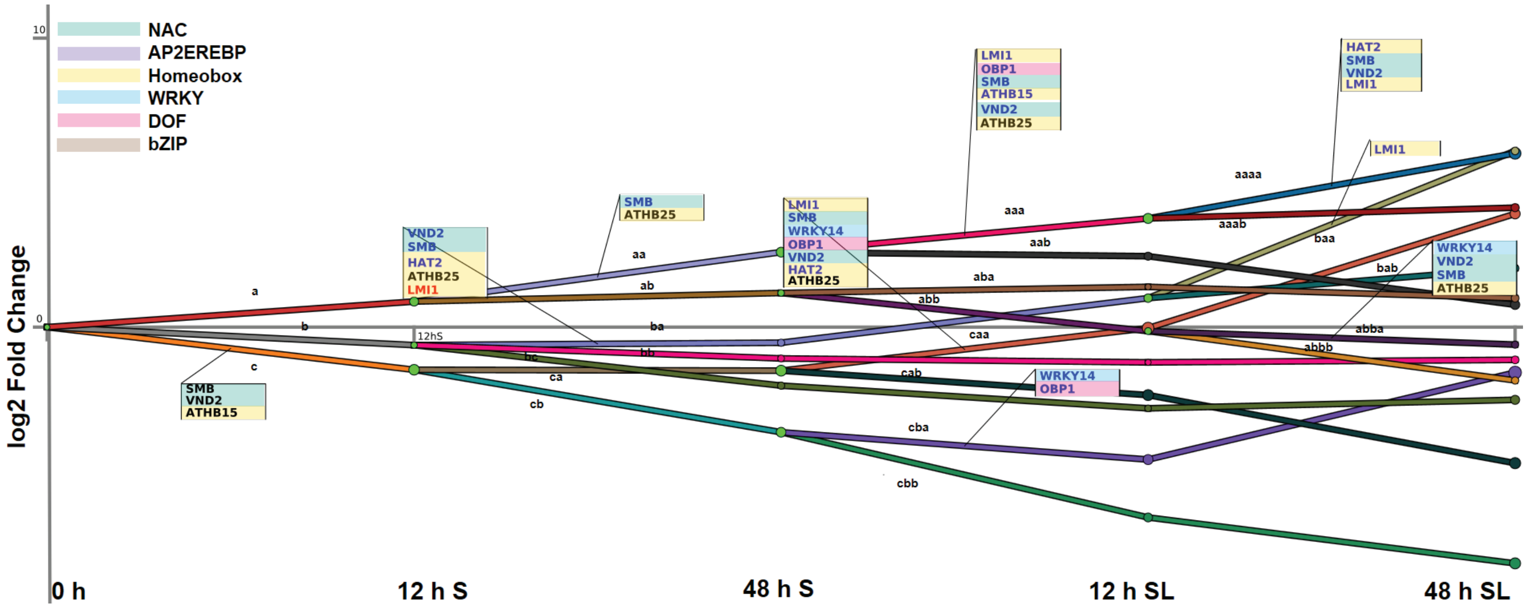

Fig. S4. Reduced DREM model showing the transcription factors selected for validation. The branches of the model are individually labelled and the number of misexpressed genes in each mutant genotype is indicated in Supp. Table 4.

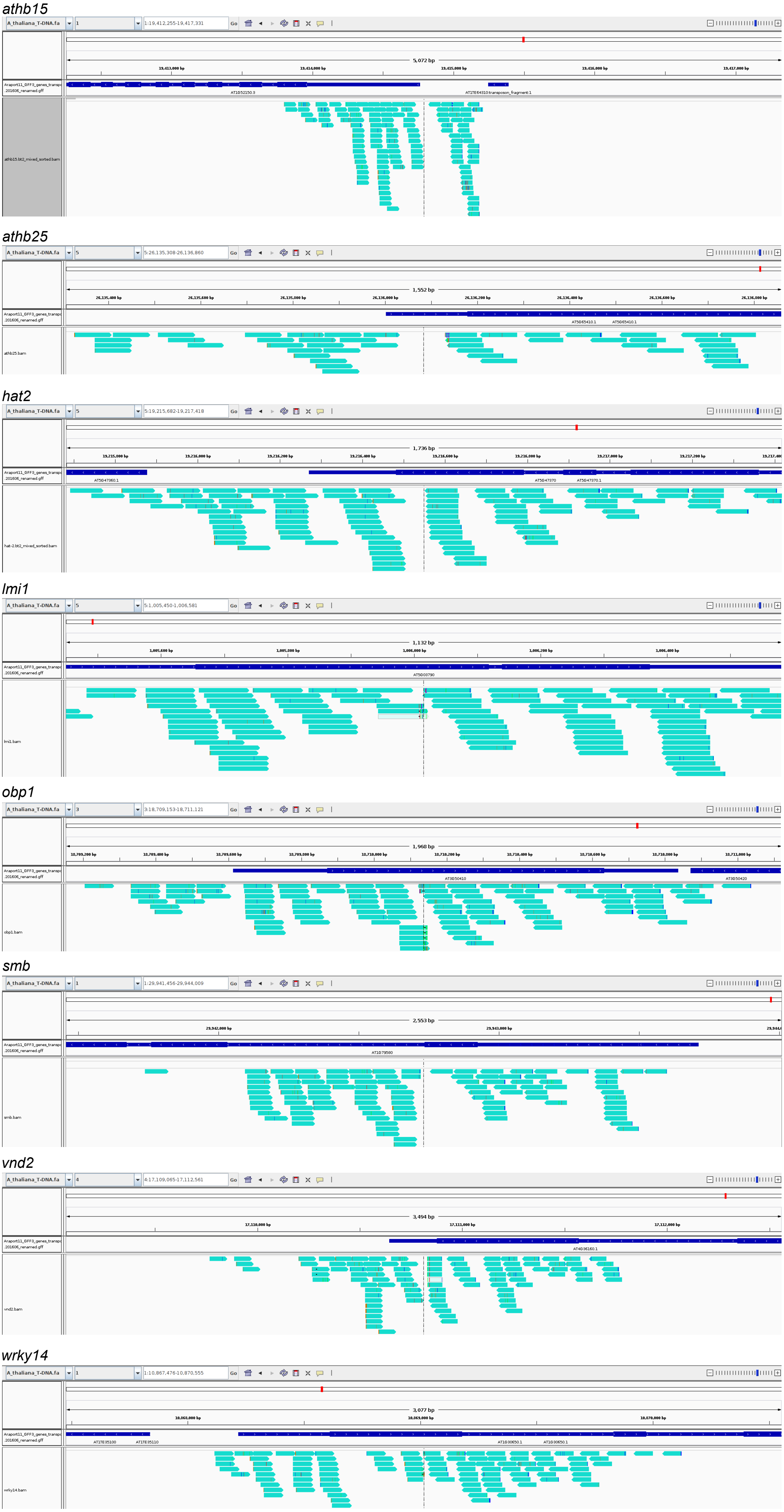

Fig. S5 Results of the whole-genome resequencing of the eight mutant lines of DREM-predicted TFs. Reads coloured in blue map to the Arabidopsis genome at the position shown but have their mate read mapping to the T-DNA sequence. Only one such insertion was found in each of the mutant lines, at the predicted position: For athb15 (SALK_140350C), in the immediate promoter of AT1G52150 between 19,414,788 and 19,414,820 bp; for athb25 (SALK_133857C), in the 5' UTR of AT5G65410 around 26,136,131 bp; for hat2 (SALK_091887C), in the last exon of AT5G47370 at 19,216,555 bp; for lmi1 (SALK_131946C), in the second and exon of AT5G03790 at 1,006,016 bp; for obp1 (SALK_049540C), in the fist exon of AT3G50410 at 18,710,142 bp; for smb (SALK_143526C), towards the 5' end of AT1G79580 between 29,942,720 and 29,942,742 bp; for vnd2 (SALK_026864C), in the 3' UTR of AT4G36160 between 17,110,800 and 17,110,832 bp; and for wrky14 (SALK_105170C), in the first exon of AT1G30650 at 10,869,016 bp. No such read pairs were found in resequencing wild-type Col-0.

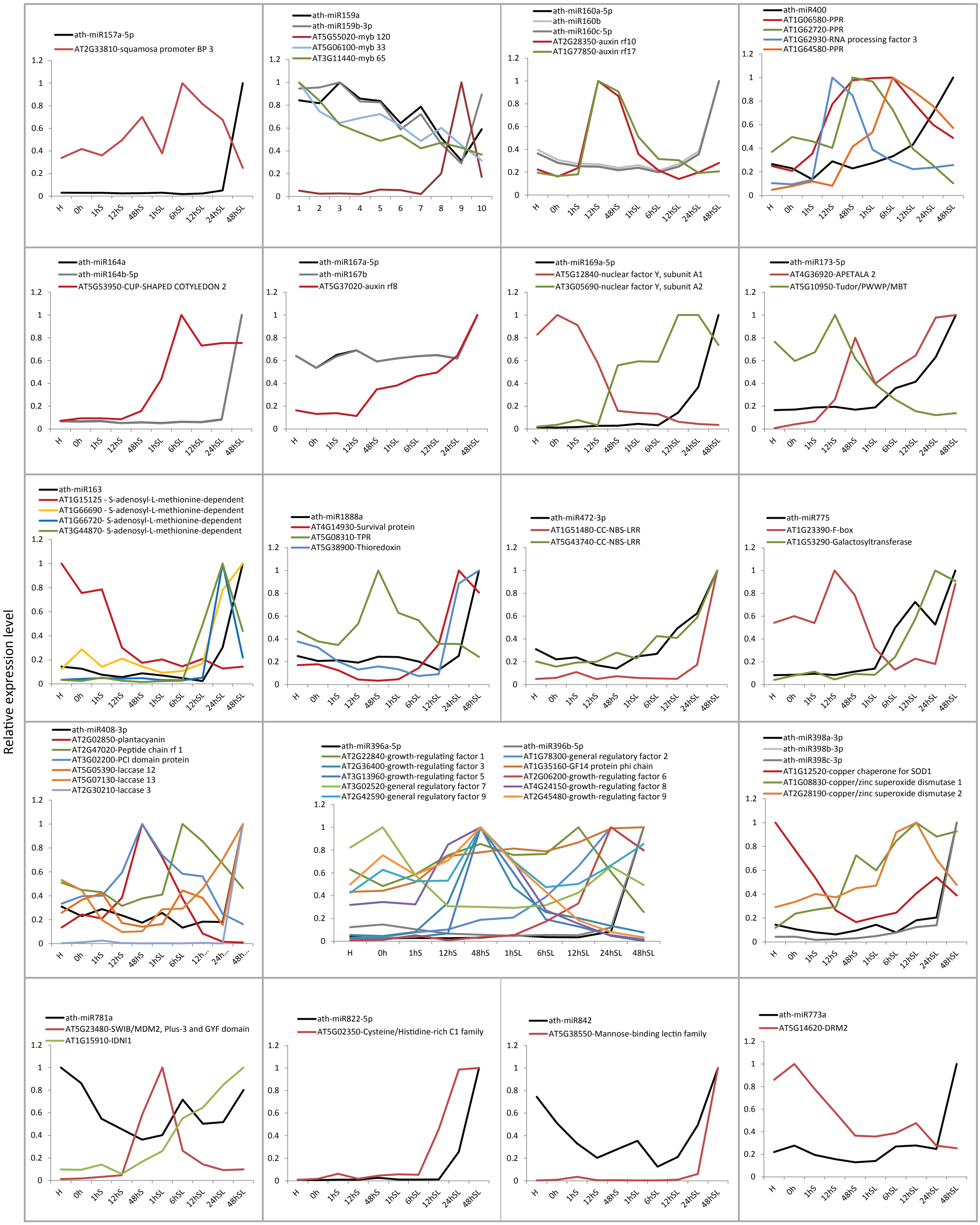

Fig. S6 Differential expression of miRNAs over seed germination. Expression profiles of all the miRNAs with known experimentally validated genes, that were differentially regulated during seed germination.

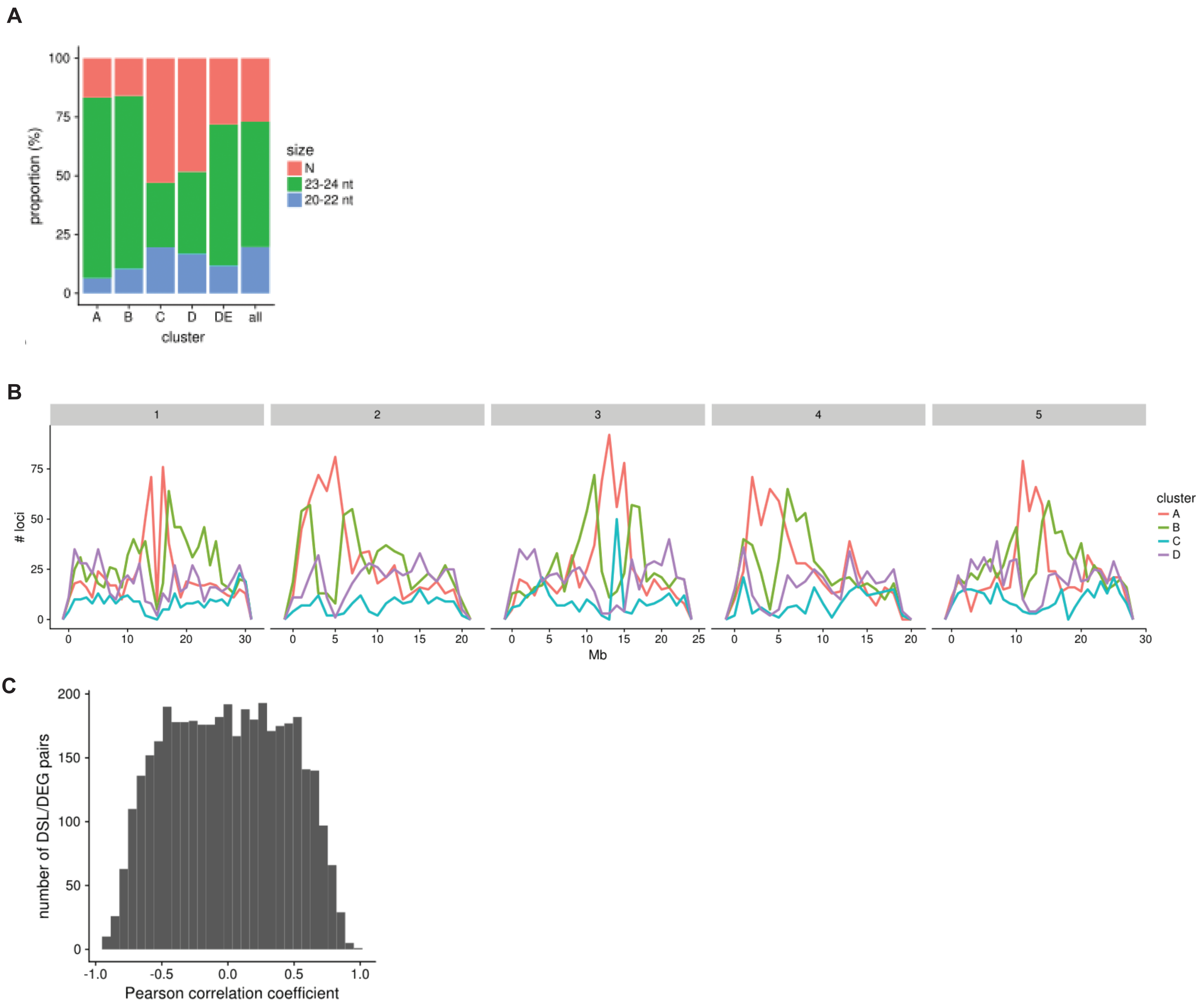

Fig. S7 (A) Dominant sRNA size class in the four clusters, differentially expressed loci (DE) and for all loci (all). Loci where less than 80% of the reads are in the 20–24-nt range are classed as “N”. (B) Distribution of the differential sRNA loci along the 5 Arabidopsis chromosomes (1 Mb bins). (C) Correlation between 20 – 22-nt sRNA loci and the expression of their target genes.

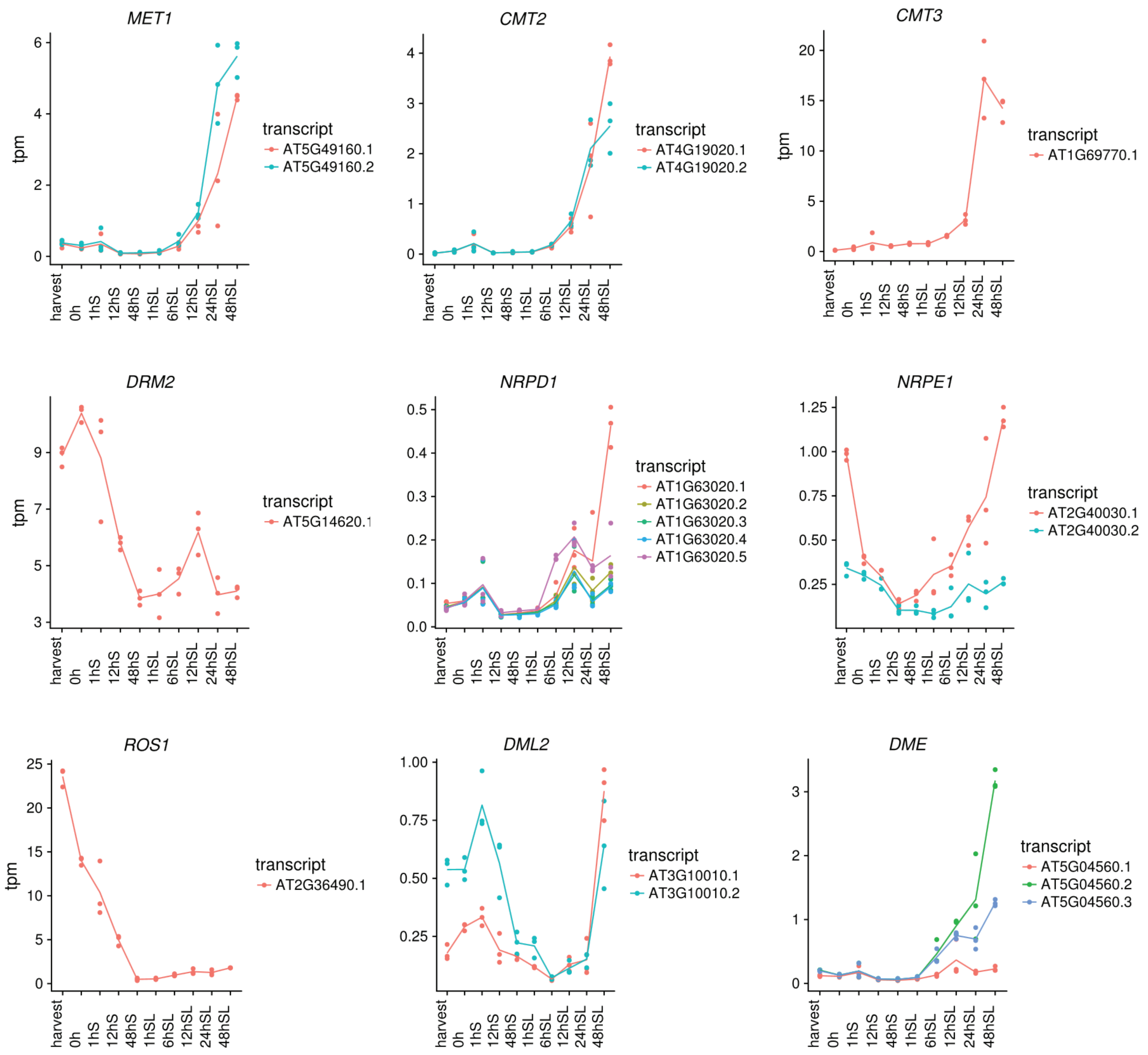

Fig. S8 Expression of the methylases, demethylases and Pol IV/V main subunits. Tpm: Transcripts per million.

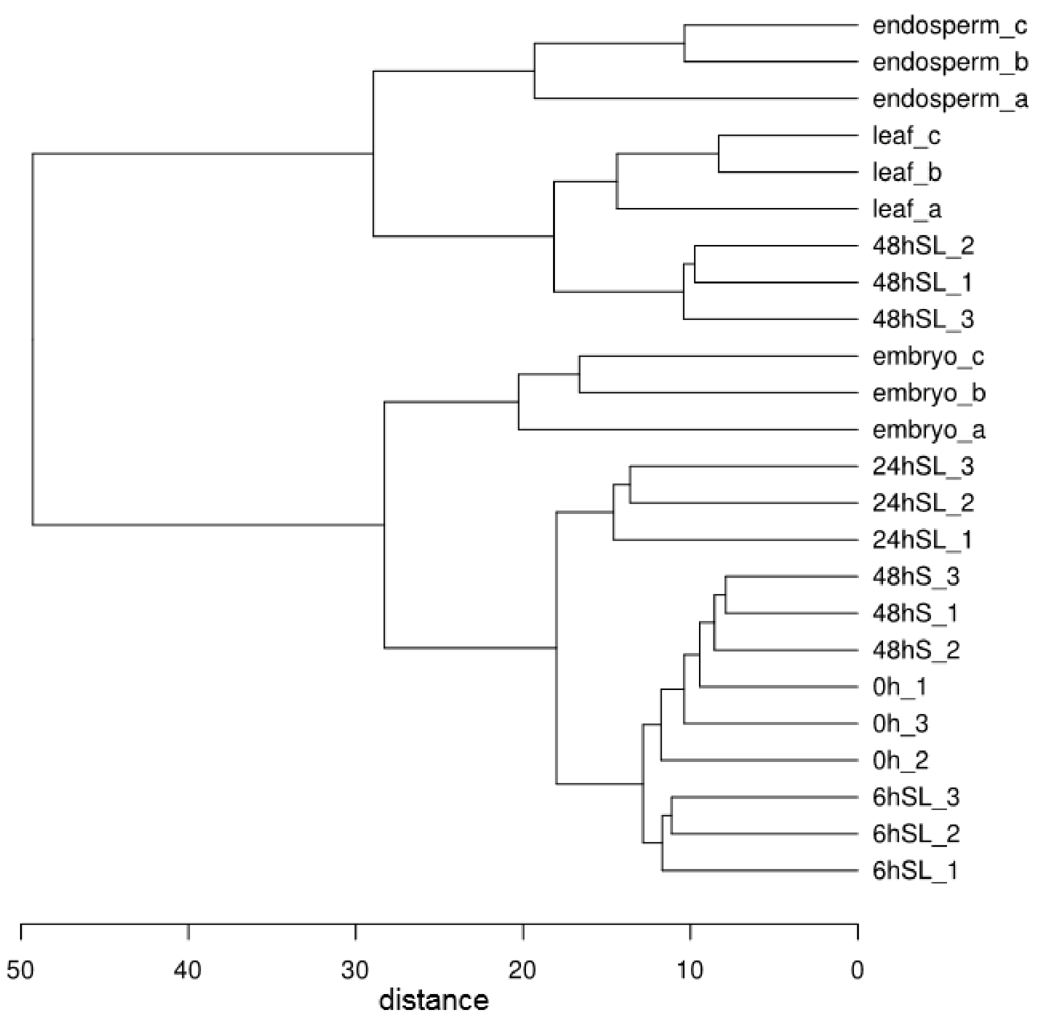

Fig S9. Hierarchical clustering of methylomes from our time course and other Arabidopsis tissues, based on the Euclidean distance of methylation levels of the 12,654 CHH DMRs, agglomerated by complete linkage. Labels: embryo_a and endosperm_a are from 7-9 days after pollination (DAP) Col-0 samples (Hsieh2009, GSE15922). Embryo_b and endosperm_b correspond to 7-8 DAP Col x Ler samples, and embryo_c and endosperm_c derive from the reciprocal Ler x Col cross (Ibarra2012, GSE38935). Leaf_a, b and c are 3-week old Col-0 leaf samples (Stroud2014, GSE39901, SRR534177, SRR534193, SRR2056571-666).

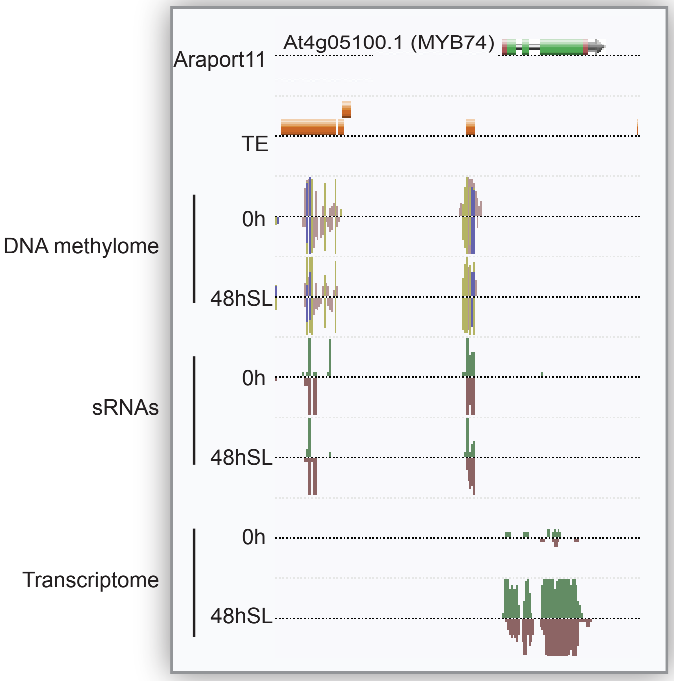

Fig. S10 AtMYB74 during seed germination. The corresponding nearby differential methylation and sRNAs in the promoter region is seen, as well as the RNA expression for this gene in the AnnoJ genome browser. Note, only the 0 h and 48 h SL samples are shown.
